# Developmental elimination of electrical synapses by UNC-51/UNC-76–mediated vesicular transport

**DOI:** 10.1083/jcb.202602115

**Published:** 2026-11-06

**Authors:** Hao Huang, Yuzhi Yang, Yuanhao Xu, Yuli Jian, Shujie Qiu, Zihao Zhao, Dong Yan, Lingfeng Meng

**Affiliations:** 1 Department of Neurology of Second Affiliated Hospital, Centre for Cellular Biology and Signaling and Cluster for Organogenesis & Homeostasis, International Joint Center for Biomedical Sciences, Zhejiang University-University of Edinburgh Institute, Zhejiang University School of Medicine, Zhejiang University, Hangzhou, China; 2 Department of Molecular Genetics and Microbiology, Duke University Medical Center, Durham, NC, USA; 3 Edinburgh Medical School, College of Medicine and Veterinary Medicine, The University of Edinburgh, Edinburgh, UK

## Abstract

Neural circuit maturation requires precise elimination of transient synaptic connections, but how electrical synapses are eliminated remains unknown. Using *Caenorhabditis elegans* PLM mechanosensory neurons, we found that gap junctions form transiently during early development and are subsequently eliminated as the circuit matures. These transient electrical synapses drive high-frequency calcium oscillations; optogenetic ablation of early gap junctions abolishes calcium dynamics and disrupts chemical synapse formation, while restoring calcium activity independently of gap junction coupling substantially rescues this defect, directly linking electrical synapse–driven calcium dynamics to chemical synapse formation. Through forward genetic screening, we identified the conserved kinase UNC-51/ULK as essential for elimination. UNC-51 phosphorylates the kinesin adaptor UNC-76/FEZ to promote RAB-10–dependent net retrograde removal of innexins, progressively depleting innexin proteins from the synaptic site. Disrupting this pathway prevents elimination, causing persistent electrical synapses and neuronal hyperactivity. These findings establish a conserved framework for developmental elimination of functionally essential transient electrical connectivity.

## Teaser

Huang et al. show that transient electrical synapses in developing *Caenorhabditis elegans* neurons generate calcium oscillations required for circuit maturation, and identify a conserved UNC-51-UNC-76-RAB-10 trafficking pathway that eliminates these synapses once this function is fulfilled, revealing a general mechanism for developmental circuit refinement.

## Introduction

Neural circuit maturation relies on the precise elimination of transient synaptic connections to achieve functional specialization (Katz and Shatz, 1996). While mechanisms governing the pruning of chemical synapses are well characterized—including activity-dependent processes such as complement-mediated tagging and microglial phagocytosis (Hegde and DiAntonio, 2002; Stevens et al., 2007; Paolicelli et al., 2011; Schafer et al., 2012; Ertürk et al., 2014; Meng et al., 2015; Stavoe and Holzbaur, 2019)—how electrical synapses are eliminated during development remains fundamentally unknown.

Electrical synapses, formed by connexin/innexin gap junction channels that create intercellular pores permeable to ions, calcium, and second messengers (Giepmans, 2004; Phelan, 2005), enable not only rapid electrical signal transmission but also direct intercellular chemical signaling. These channels exhibit remarkably rapid turnover (1–5 h) (Laird, 2006)—among the fastest in the proteome—due to continuous cycles of membrane insertion and removal (Gaietta et al., 2002; Lauf et al., 2002). This dynamic trafficking enables precise regulation of electrical coupling strength, which is directly determined by the density of gap junctions at intercellular junctions (Harris, 2001; Bukauskas and Verselis, 2004). While activity-dependent transcriptional mechanisms can modulate expression levels (Bhattacharya et al., 2019), the trafficking machinery itself provides an additional layer of control over synaptic connectivity. However, whether organisms actively exploit this trafficking machinery to programmatically adjust intercellular communication during development remains largely unexplored.

Transient electrical synapses are a conserved and widespread feature of developing neural circuits, representing the earliest functional connections to form across systems from invertebrates to mammals (Blackshaw and Warner, 1976; Peinado et al., 1993; Dupont et al., 2006). During this early developmental window, they serve remarkably diverse functions: coordinating left–right asymmetry in *Caenorhabditis elegans* AWC neurons (Chuang et al., 2007); driving spontaneous activity waves for circuit refinement in the mammalian retina (Blankenship and Feller, 2010); and synchronizing spinal interneuron networks to drive the earliest embryonic movements in zebrafish (Saint-Amant and Drapeau, 2001). These early electrical connections are not merely active during this window—they are functionally required for the formation of the chemical synapses that will eventually replace them: disrupting gap junction expression in leech neurons prevents chemical synaptogenesis (Todd et al., 2010); blocking electrical coupling between sister pyramidal cells in mouse cortex impairs their later chemical connectivity (Yu et al., 2012); loss of connexin/innexin-based gap junctions disrupts mature chemical synapse formation in mouse olfactory bulb, ferret visual cortex, and *Drosophila* optic lamina (Kandler and Katz, 1998; Curtin et al., 2002; Maher et al., 2009); and loss of the gap junction protein Gjd2b in zebrafish cerebellum reduces glutamatergic synapse density on Purkinje’s neurons (Sitaraman et al., 2021). Although gap junctions are known to transmit calcium and second messengers, whether these signals causally instruct chemical synapse development—and how these transiently essential connections are themselves subsequently eliminated—remains mechanistically unclear.

To address these questions, we turned to *C. elegans* PLM mechanosensory neurons, which form electrically coupled synapses with multiple interneuron partners—including PVC, LUA, PVR, and BDU—as defined by electron micrographic reconstruction (White et al., 1986; Zhang et al., 2013), and express a combination of innexins including UNC-9, INX-1, and UNC-7 that can assemble into molecularly distinct heterochannel configurations at individual synapses (Walker and Schafer, 2020; Vats et al., 2026). Using this system, we show that transient electrical synapses coordinate high-frequency calcium oscillations during early larval development, and that loss of these synapses abolishes calcium dynamics and disrupts chemical synapse formation—directly linking electrical coupling, calcium signaling, and chemical synaptogenesis within a single system. Through forward genetic screening, we identify the conserved kinase UNC-51/ULK as the central regulator of electrical synapse elimination, acting through a phosphorylation-dependent trafficking mechanism that drives directional removal of innexin proteins from synapses. These findings define the developmental function of transient electrical synapses in instructing circuit assembly, and uncover a conserved kinase-driven trafficking mechanism that eliminates them once this function is fulfilled.

## Results

### Electrical synapses undergo developmental elimination in mechanosensory neurons

**Figure 1.**
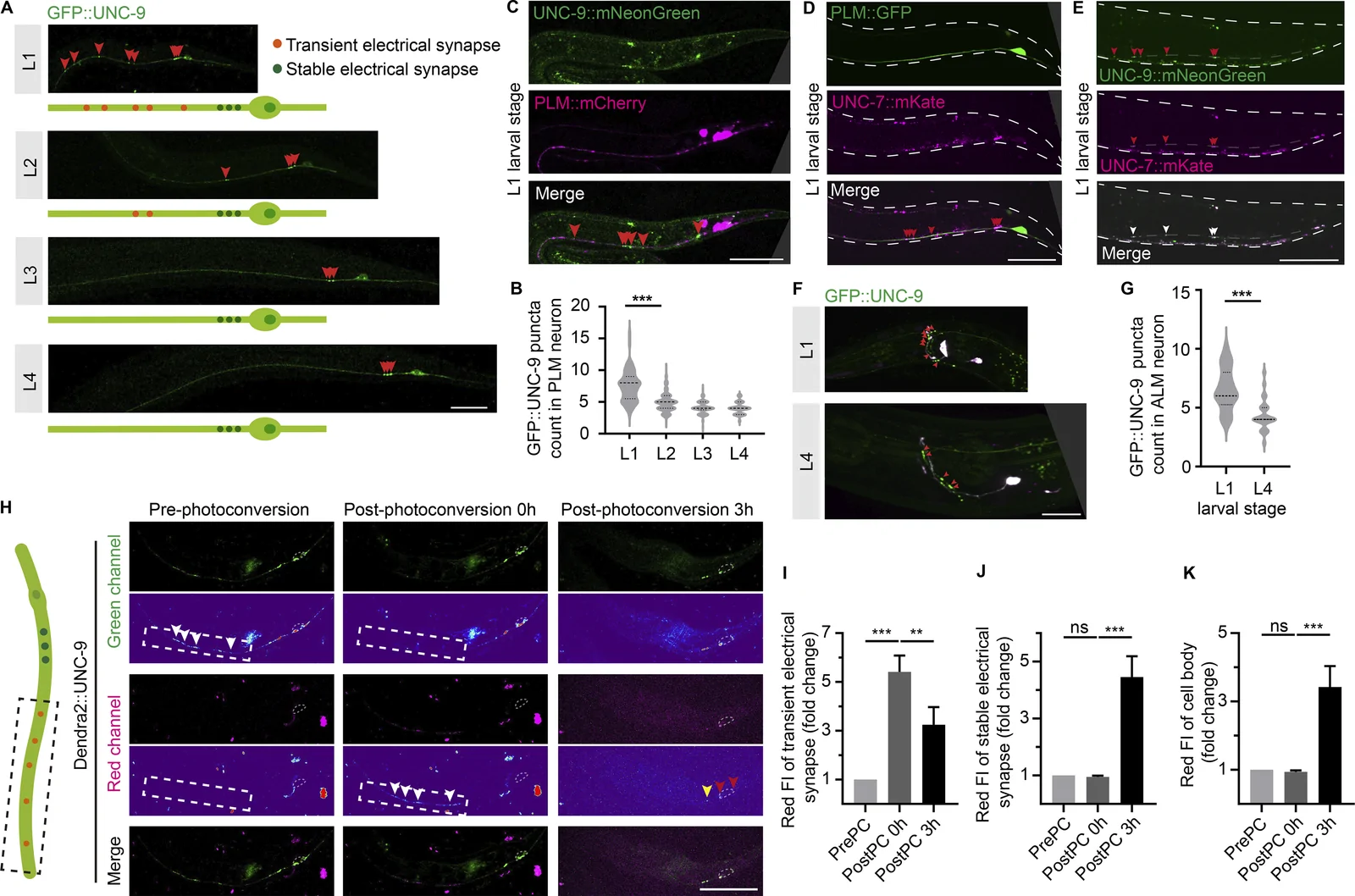
**Transient electrical synapses are developmentally eliminated in mechanosensory neurons. (A)** Representative fluorescence images of GFP::UNC-9-labeled electrical synapses in PLM neurons across larval development (L1, L2, L3, and L4) in *yadIs12* animals. Red arrowheads indicate electrical synapses in the confocal image, while the schematic figure illustrates the elimination of PLM electrical synapses from L1 to L4. **(B)** Quantification of GFP::UNC-9–labeled electrical synapses in PLM neurons across larval development in *yadIs12* animals. *n* > 100 per stage. The dashed lines represent the quartiles and median of the data distribution. **(C)** Representative fluorescence images of endogenously C-terminally tagged UNC-9::mNeonGreen and *mec-4* promoter–driven mKate2 in PLM neurons at the L1 stage. Red arrowheads indicate electrical synapses. **(D)** Representative fluorescence images of endogenously C-terminally tagged UNC-7::mKate and *mec-4* promoter–driven GFP in L1-stage PLM neurons. Red arrowheads indicate electrical synapses. **(E)** Representative fluorescence images of UNC-9::mNeonGreen and UNC-7::mKate colocalization in PLM neurons at the L1 stage. Red arrowheads indicate electrical synapses, and white arrowheads indicate electrical synapses showing colocalization of UNC-9 and UNC-7. The semi-transparent dashed line outlines the PLM axon. **(F)** Representative fluorescence images of GFP::UNC-9–labeled electrical synapses in ALM neurons at L1 and L4 in *yadIs12* animals. Red arrowheads indicate electrical synapses. **(G)** Quantification of GFP::UNC-9–labeled electrical synapses in ALM neurons at L1 and L4 stages in *yadIs12* animals. *n* = 20 (L1), *n* = 24 (L4). **(H)** Representative images of photoconvertible Dendra2::UNC-9 on PLM before photoconversion, immediately after photoconversion, and 3 h after photoconversion, shown in red and green channel, with corresponding heat map images indicating signal intensity. White, red, and yellow arrowheads indicate Dendra2::UNC-9 puncta along the PLM neurites, in the cell bodies, and at stable electrical synapses, respectively. The semi-transparent dashed line outlines the cell body. **(I–K)** Quantification of red Dendra2::UNC-9 fluorescence signal in transient/stable electrical synapse area and cell body at before photoconversion, immediately after photoconversion, and 3 h after photoconversion. *n* = 20 per condition. Scale bars, 20 μm. Data are presented as the mean ± SEM. Statistical analyses were performed using the Kruskal–Wallis test with Dunn’s test (B), Mann–Whitney test (G), Student’s *t* test (I, 0 vs 3 h), Wilcoxon signed-rank test (J and K, 0 vs 3 h), and one-sample *t* test (I–K, pre vs 0 h). ns: not significant, **P < 0.01, ***P < 0.001.

To investigate electrical synapse regulation during development, we focused on PLM mechanosensory neurons in *C. elegans*, which form electrical connections with multiple partners (White et al., 1986; Simonsen et al., 2014). Electrical synapses were visualized using the stable GFP::UNC-9 transgenic line (*yadIs12*) since UNC-9 is a major gap junction component in PLM neurons and GFP::UNC-9 puncta have been validated to mark electrical synapses (Meng et al., 2016). Quantification of electrical synapse numbers along PLM neurites across L1 (first larval stage) to L4 (fourth larval stage) revealed that L1 displayed ∼8 electrical synapses (median = 8) distributed along the entire neurite. As development progressed, electrical synapse numbers significantly decreased by L2 (median = 5) and stabilized at reduced levels during L3 and L4 (median = 4 for both stages) (Fig. 1, A and B). Notably, the most pronounced decline occurred between L1 and L2 (Fig. 1 B), demonstrating that the majority of electrical synapse elimination takes place by L2. We henceforth refer to the gap junctions eliminated between L1 and L4 as transient electrical synapses, and the subset located near the cell body that persists into L4 as stable electrical synapses.

**Figure S1.**
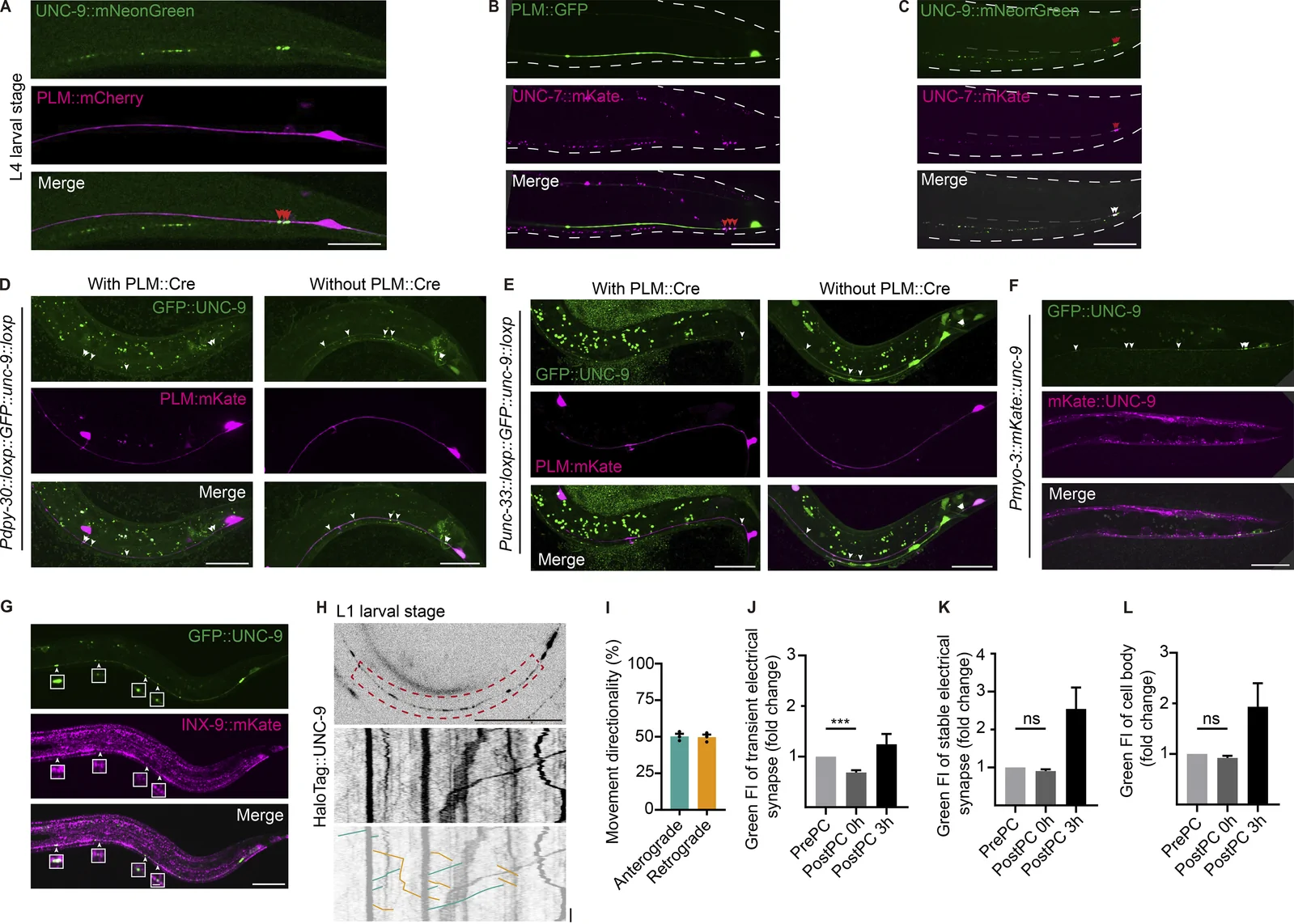
**Validation of transient electrical synapse dynamics with independent reporters.** **(A)** Representative fluorescence images of endogenously C-terminally tagged UNC-9::mNeonGreen and *mec-4* promoter–driven mKate2 in L4-stage PLM neurons. Red arrowheads indicate electrical synapses. **(B)** Representative fluorescence images of endogenously C-terminally tagged UNC-7::mKate and *mec-4* promoter–driven GFP in L4-stage PLM neurons. Red arrowheads indicate electrical synapses. **(C)** Representative fluorescence images of colocalization of UNC-9::mNeonGreen and UNC-7::mKate in PLM neurons at the L4 stage. Red arrowheads indicate electrical synapses, and white arrowheads indicate UNC-9– and UNC-7–colocalized electrical synapses. The semi-transparent dashed line traces the PLM axon. **(D)** Representative fluorescence images of L1 animals with ubiquitous GFP::UNC-9 expression driven by the *dpy-30* promoter (*Pdpy-30::loxP::GFP::unc-9::loxP*; *Pmec-4::mKate2*) with or without PLM-specific Cre expression. White arrowheads indicate residual UNC-9 puncta along the PLM axon. **(E)** Representative fluorescence images of L1 animals with pan-neuronal GFP::UNC-9 expression driven by the *unc-33* promoter (*Punc-33::loxP::GFP::unc-9::loxP*; *Pmec-4::mKate2*) with or without PLM-specific Cre expression. White arrowheads indicate residual UNC-9 puncta along the PLM axon. **(F)** Representative fluorescence images of the tail region of L1 animal co-expressing muscle-derived mKate::UNC-9 and PLM-derived GFP::UNC-9 (*Pmyo-3::mKate::unc-9; Pmec-4::GFP::unc-9*). White arrowheads indicate electrical synapses. **(G)** Representative fluorescence images of colocalized epidermal INX-9 and PLM UNC-9 puncta in L1 animal (*Pdpy-7::inx-9::mKate; Pmec-4::GFP::unc-9*). White arrowheads point to colocalized electrical synapses. **(H)** Representative time-lapse imaging of electrical synapse dynamics visualized using HaloTag::UNC-9 in WT animals. The blue and yellow lines indicate UNC-9 anterograde and retrograde movement, respectively. **(I)** Quantification of the directional movement of UNC-9 puncta originating from transient electrical synapses in PLM neurons of L1 *Pmec-4::HaloTag::unc-9* animals. *n* = 17. **(J–L)** Quantification of green Dendra2::UNC-9 fluorescence signal in transient / stable electrical synapse area and cell body at before photoconversion, immediately after photoconversion, and 3 h after photoconversion. *n* = 20 per condition. Scale bars, 20 μm for confocal images, 2 μm for inset magnifications, and 10 μm (horizontal) and 30 s (vertical) for time-lapse images; data are presented as the mean ± SEM. Statistical analyses were performed using Student’s *t* test (I) and one-sample *t* test (J–L). ns: not significant, ***P < 0.001.

For confirmation that the observed elimination pattern reflects endogenous protein dynamics rather than transgenic artifacts, CRISPR-mediated endogenous tagging of UNC-9 with mNeonGreen was performed. Endogenously tagged UNC-9::mNeonGreen in PLM neurons showed a similar developmental decline from L1 to L4 (Fig. 1 C and Fig. S1 A), validating our findings from the transgenic line. We note, however, that the UNC-9::mNeonGreen knock-in strain displays classic *unc-9* mutant defects, including kinker locomotion (unpublished observations), raising the possibility that this tag partially compromises UNC-9 function and its subcellular localization. To corroborate our findings with an independent and functionally normal reporter, and to further characterize the molecular composition of transient electrical synapses, we generated an endogenously tagged UNC-7::mKate knock-in strain, which showed no detectable behavioral abnormalities (unpublished observations). UNC-7 similarly formed transient puncta along PLM neurites at L1 that were subsequently eliminated by L4 (Fig. 1 D and Fig. S1 B). Moreover, colocalization analysis revealed that UNC-9 and UNC-7 puncta significantly overlapped at stable gap junctions in L4 animals (Fig. S1 C), consistent with previous reports of UNC-7/UNC-9 heterochannel formation in PLM neurons (Starich et al., 2009). Similar colocalization was observed at transient electrical synapses in L1 (Fig. 1 E), suggesting that these transient junctions are likewise heteromeric channels containing both innexin subunits.

Because innexins can function as gap junction and unpaired hemichannels (Phelan et al., 2008; Scemes et al., 2009; Holcroft et al., 2013; Burendei et al., 2020; Nakayama et al., 2024), and light microscopy alone cannot resolve whether a partnering cell contributes, we tested whether the transient UNC-9 puncta in PLM neurons reflect a contribution from another cell. We generated a *Pdpy-30::loxP::GFP::unc-9::loxP* transgene combined with PLM-specific Cre recombinase expression to selectively remove UNC-9 from PLM neurons. Despite cell-autonomous excision of UNC-9 in PLM, GFP::UNC-9 puncta were still observed along PLM axons (Fig. S1 D), indicating that these puncta arise from innexin expressed in a partnering cell rather than from PLM neurons alone, and supporting the interpretation that transient electrical synapses in PLM represent genuine intercellular gap junctions rather than hemichannels.

Having established that these transient junctions involve a partnering cell, we next sought to identify its identity. We considered the anatomical context of PLM neurons during L1 development. Electron microscopic reconstruction has shown that PLM neurons lie in close proximity to neurons, body muscle, and hypodermal cells during this period (White et al., 1986; Bonacossa-Pereira et al., 2022), raising these as candidate partner cell types. To distinguish among them, we tested each tissue for colocalization with PLM’s transient UNC-9 puncta using the innexin predominantly expressed there: UNC-9, via a pan-neuronal *Punc-33::loxP::GFP::unc-9::loxP* transgene combined with PLM-specific Cre expression, for neurons (Fig. S1 E); UNC-9 for body muscle (Fig. S1 F); and INX-9 for the hypodermis (Bhattacharya et al., 2019) (Fig. S1 G). Partial colocalization was observed between PLM UNC-9 puncta and hypodermal INX-9 (Fig. S1 G), raising the possibility that hypodermal cells may represent transient gap junction partners of PLM neurons.

To determine whether this phenomenon extends beyond PLM neurons, we examined ALM mechanosensory neurons, which form electrical connections with AVD interneurons in the nerve ring through UNC-9 and UNC-7 innexins (Jin et al., 2020; He et al., 2026). Using the *yadIs12* transgenic reporter, we quantified UNC-9 puncta in ALM across development. At L1, ALM neurons displayed approximately seven puncta (median = 6), which significantly decreased to approximately four puncta (median = 4) by L4 (Fig. 1, F and G). Together, these results demonstrate that electrical synapse elimination is not restricted to PLM neurons, but represents a conserved developmental process in *C. elegans* mechanosensory neurons.

Gap junctions are known to undergo rapid, continuous turnover (Meng et al., 2016). We first asked whether transient synapses similarly exhibit this dynamic trafficking behavior, raising the question of whether transient electrical synapses retain the capacity for retrograde transport toward the soma. We first examined the general dynamic trafficking behavior of transient synapses using time-lapse imaging of HaloTag::UNC-9 in L1 PLM neurons. This analysis revealed that UNC-9 puncta originating from transient electrical synapses moved bidirectionally along the neurite, with comparable frequencies of anterograde and retrograde movement (Fig. S1, H and I), resembling the bidirectional trafficking pattern of stable electrical synapses in young adult animals (Meng et al., 2016). These observations suggested that transient electrical synapses also exhibit highly dynamic properties. To further validate the dynamic behavior of electrical synapses and quantify protein turnover rates, photoactivation experiments were performed using photoconvertible Dendra2::UNC-9 in L1 PLM neurons. Selective photoactivation of Dendra2::UNC-9 at transient electrical synapses induced a conversion from green to red fluorescence (boxed region, Fig. 1 H), and the red fluorescent signal was monitored at 0 and 3 h after activation. Following photoactivation, green fluorescence at the targeted region decreased by half, whereas red fluorescence increased approximately sixfold compared with the preactivation baseline, indicating efficient photoconversion (Fig. 1 I and Fig. S1 J). The fluorescence signal at stable electrical synapses and the soma remained unchanged (Fig. 1, J and K; and Fig. S1, K and L), demonstrating specificity of the photoactivation. Over the following 3 h, red fluorescence at transient electrical synapses declined by half (Fig. 1 I). However, red fluorescence increased at stable gap junctions (approximately fourfold) and in the cell body (approximately threefold) relative to preactivation baseline (Fig. 1, J and K). This turnover rate closely matches that of stable junctions (Meng et al., 2016), demonstrating that transient synapses retain the capacity for retrograde transport toward the soma.

### Transient electrical synapses coordinate calcium dynamics to regulate chemical synapse formation

**Figure 2.**
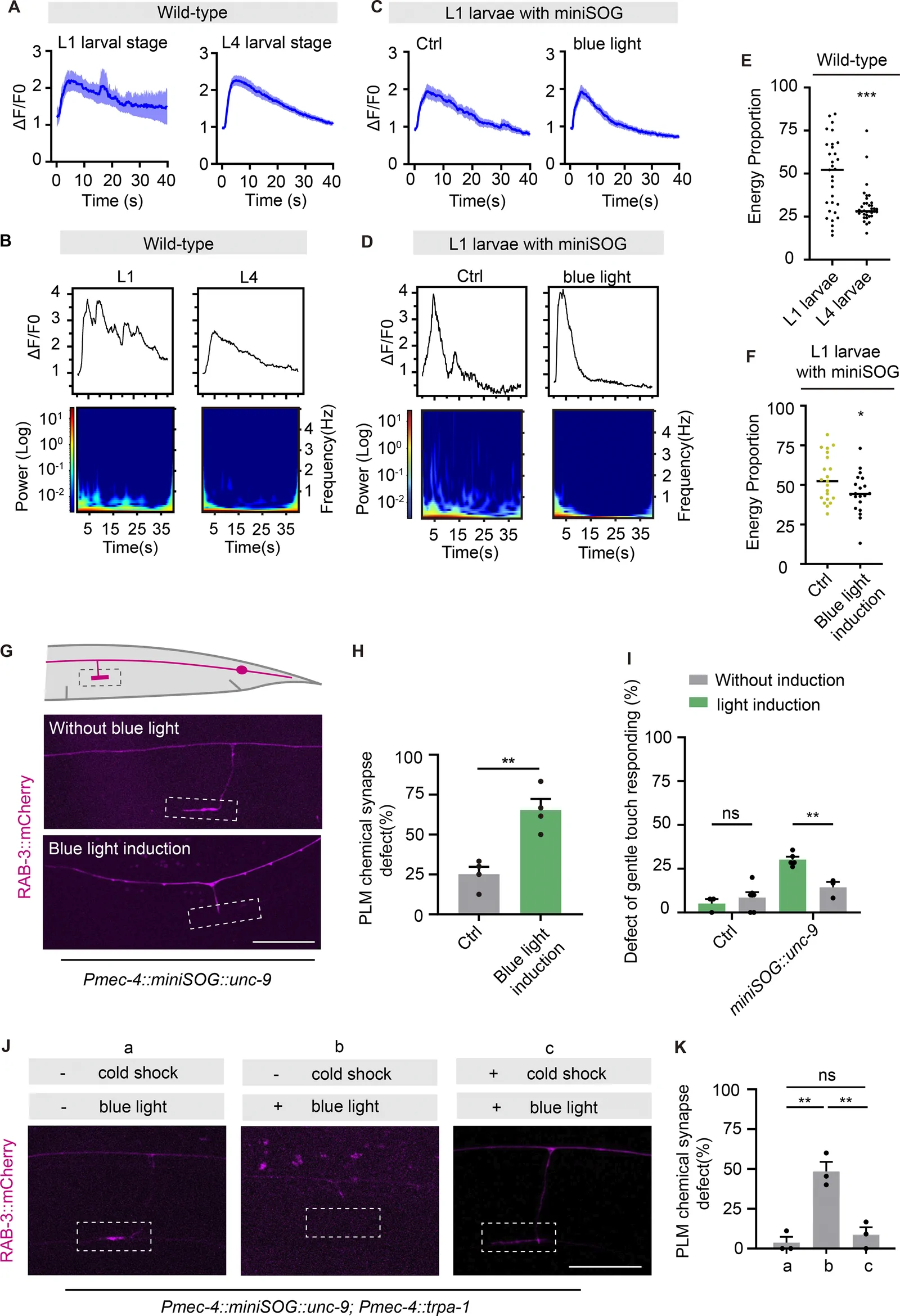
**Transient electrical synapses coordinate calcium dynamics and regulate chemical synapse formation. (A)** Population-averaged calcium trace of PLM neurons in L1 and L4 GCaMP6-expressing WT background animals. Blue line and shading represent the mean ± SEM. *n* > 30 per stage. **(B)** Representative PLM calcium trace and wavelet spectrogram in L1 and L4 WT background animals expressing *mec-7* promoter–driven GCaMP6. Calcium activity was quantified as changes in GCaMP fluorescence normalized to baseline (ΔF/F_0_). **(C)** Population-averaged PLM calcium trace of L1 *Pmec-4::mini-SOG::unc-9* GCaMP6-expressing animals with or without blue light activation. *n* = 21(Ctrl), *n* = 20 (blue light). **(D)** Representative PLM calcium trace and wavelet spectrogram in L1 *Pmec-4::miniSOG::unc-9* GCaMP6-expressing *C. elegans* with or without blue light activation. **(E)** Quantification of the secondary calcium signal energy proportion of PLM neuron in L1 and L4 GCaMP6-expressing WT background animals. The secondary calcium signal was defined as the ratio of summed energy of components >0.1 Hz during the secondary response window (5–30 s) to that during the full response period (2–38 s). The lines represent the median of data distribution. *n* > 30 per stage. **(F)** Quantification of the secondary calcium signal energy proportion of PLM neurons in L1 *Pmec-4::miniSOG::unc-9* GCaMP6-expressing animals with or without blue light activation. *n* = 21(Ctrl), *n* = 20 (blue light). **(G)** Representative images of PLM chemical synaptic branch in L4 animals expressing miniSOG::UNC-9 and *mec-7* promoter–driven RAB-3::mCherry. The PLM chemical synapse was present without blue light induction but was absent after blue light induction. The schematic figure illustrates the normal PLM chemical synaptic. The dashed box highlights the chemical synapse position. **(H)** Quantification of PLM chemical synapse defects in L4 animals expressing RAB-3::mCherry and miniSOG::UNC-9 with or without blue light induction. *n* = 38 (without induction) and *n* = 40 (with induction). **(I)** Quantification of gentle touch response defects in L4-stage control animals and animals expressing miniSOG::UNC-9 with or without blue light induction. *n* > 34 per condition. **(J and K)** Representative images of PLM chemical synaptic branches and quantification of chemical synapse defects in L4 animals expressing PLM-specific miniSOG::UNC-9 and TRPA-1 under different treatment conditions: (a) no cold shock and no blue light induction; (b) no cold shock with blue light induction; (c) blue light induction followed by cold shock. For condition c, L1 animals were first subjected to blue light to activate the optogenetic system, followed by cold shock treatment. After a 27-h recovery period, animals were analyzed at the L4 stage. *n* = 27 (a), 31 (b), 26 (c). Scale bars, 20 μm. Data are presented as the mean ± SEM. Statistical analyses were performed using Student’s *t* test with Welch’s correction (E), Student’s *t* test (F, I, and H), and one-way ANOVA with Tukey’s test (K). ns: not significant, *P < 0.05, **P < 0.01, ***P < 0.001.

To determine the functional role of transient electrical synapses, we next examined spontaneous calcium activity in PLM neurons at L1 (transient electrical synapses present) and L4 (transient electrical synapses eliminated) using GCaMP6. Both L1 and L4 animals exhibited typical PLM calcium responses to mechanical stimulation (Fig. 2 A), confirming that sensory function is intact across developmental stages. However, wavelet transform analysis of calcium dynamics revealed that L1 neurons exhibited significantly higher energy proportion (48.8%) compared with L4 (31.0%) (Fig. 2, B and E). This metric captures the fraction of high-frequency (>0.1 Hz) activity that persists beyond the initial stimulus-evoked transient (see Materials and methods), such that a higher value reflects sustained oscillatory fluctuations rather than a single clean response—the expected signature of ongoing electrical synapse–mediated network activity, raising the possibility that electrical synapses contribute to the enhanced high-frequency oscillations seen during early development.

We then assessed whether electrical synapses are required for these oscillations by performing optogenetic ablation using miniSOG-tagged UNC-9, and selectively disrupting L1 transient electrical synapses by restricting blue light illumination to their location. While basal calcium signals remained unchanged (Fig. 2 C), indicating that overall PLM neuron function was preserved after blue light treatment, wavelet analysis revealed that optogenetic ablation significantly reduced high-frequency calcium activity (from 54.0% to 44.6%) (Fig. 2, D and F), confirming that transient electrical synapses mediate coordinated high-frequency calcium dynamics during early larval development.

**Figure S2.**
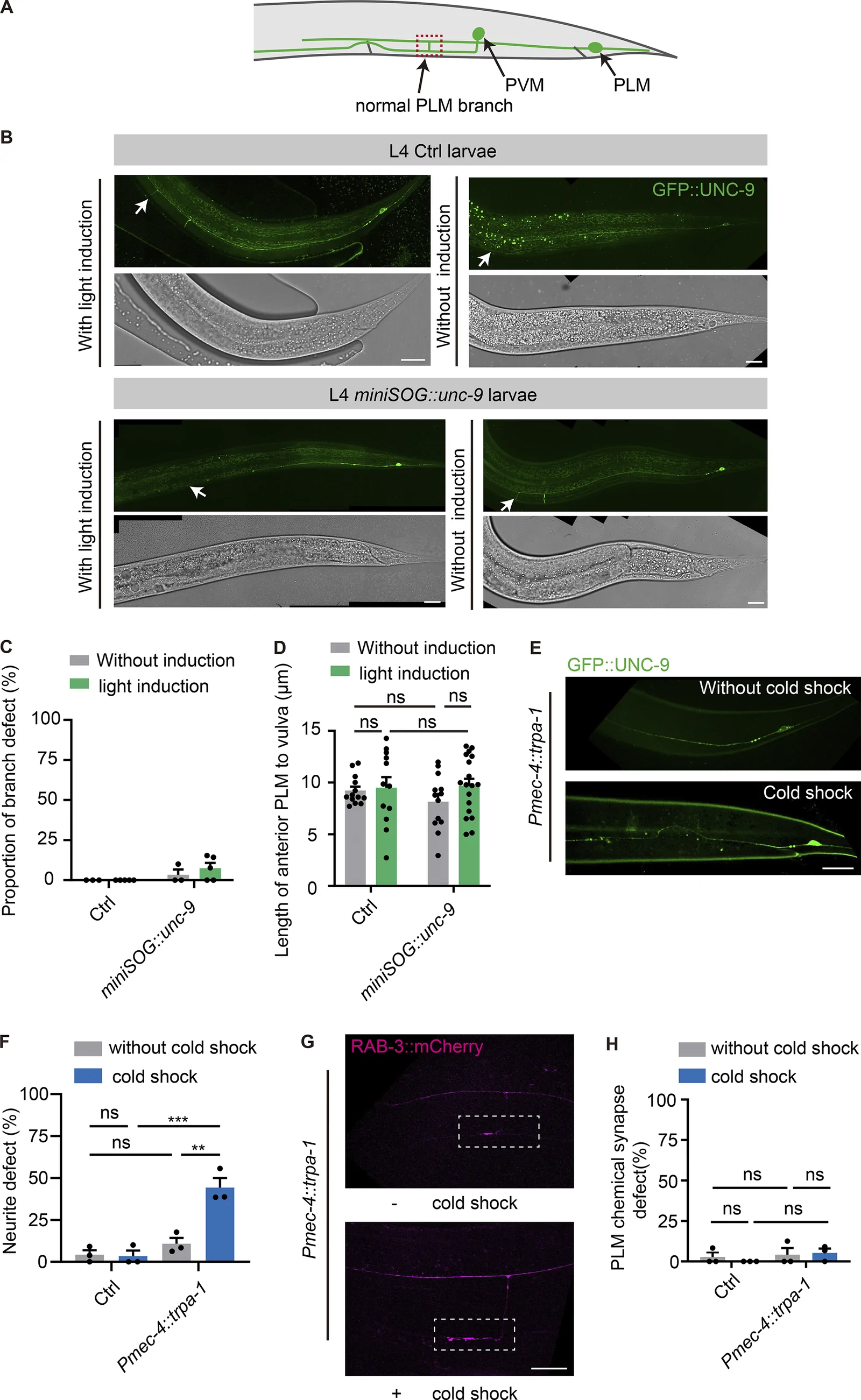
**Controls for neurite morphology and chemical synapse assays.** **(A)** Schematic figure of WT PLM neuron anterior axon morphology, showing a branch extending toward PVM and the axon terminus near the vulva. The dashed box highlights the PLM branch. **(B)** Representative fluorescence and DIC images showing PLM anterior axon branches in L4 control and *Pmec-4:: miniSOG::unc-9* animals with or without blue light induction. White arrowheads point to axon branches. **(C and D)** Quantification of excessive axon branch defects (C) and the distance from the anterior PLM axon terminus to the vulva (D) in L4 *Ctrl* and *Pmec-4:: miniSOG::unc-9* animals with or without blue light induction. For figure C, *n* > 24 per group. For figure D, *n* ≥ 12 per group. **(E and F)** Representative images of PLM neurites (E) and quantification of PLM neurite defects (F) in L4 animals expressing PLM-specific TRPA-1 with or without cold shock treatment. *n* ≥ 34 per condition. **(G and H)** Representative images of PLM chemical synaptic branches (G) and quantification of chemical synapse defects (H) in L4 animals expressing PLM-specific TRPA-1 with or without cold shock treatment. *n* ≥ 24 per condition. Scale bars, 20 μm. Data are presented as the mean ± SEM. Statistical analyses were performed using one-way ANOVA (H and F) and two-way ANOVA (C and D) with Tukey’s test. ns: not significant, **P < 0.01, ***P < 0.001.

To assess the biological significance of these electrical synapse–mediated calcium dynamics, we first examined neurite outgrowth and overall neurite length, but found no significant morphological defects (Fig. S2, A and B). We next examined chemical synapse formation, given that electrical synapses regulate chemical synaptogenesis in many systems (Jabeen and Thirumalai, 2018) and PLM chemical synapses mature primarily during L1 (Xu and Quinn, 2016). Analysis of chemical synapse formation using the presynaptic marker RAB-3 revealed striking defects. Control animals expressing miniSOG-tagged UNC-9 without blue light exposure displayed chemical synapse abnormalities in ∼25% of PLM neurons (Fig. 2, G and H). In contrast, optogenetic ablation dramatically increased this proportion to 65% (Fig. 2 H), with many neurons exhibiting the complete absence of RAB-3 puncta at expected synaptic locations (Fig. 2 G). These findings indicate that transient electrical synapses during L1 are critical for proper chemical synapse formation, and their premature elimination disrupts the establishment of mature chemical connectivity.

We next assessed whether these structural and functional deficits translate into behavioral abnormalities by examining posterior mechanosensory responses (gentle touch) in animals subjected to miniSOG-mediated ablation of transient electrical synapses at L1. At L4, ablated animals exhibited a significantly higher proportion of touch response abnormalities compared with nonablated controls (Fig. 2 I), demonstrating that transient electrical synapses are required for mechanosensory behavior.

To further examine the relationship between calcium activity and electrical synapse–mediated chemical synapse formation, we used a rescue strategy to experimentally restore calcium oscillations after electrical synapse ablation. We expressed the temperature-gated channel TRPA-1 in PLM neurons (*Pmec-4::trpa-1*), which confers cold-induced high-frequency calcium activity independent of gap junction coupling (Chatzigeorgiou et al., 2010). To validate this line prior to the rescue experiments, we characterized PLM neuron morphology and chemical synapse structure in unablated animals with and without cold stimulation. The transgene alone caused no abnormalities in either (Fig. S2, F–H). Cold stimulation, however, induced overt morphological abnormalities in ∼50% of animals, while chemical synapse formation remained unaffected (Fig. S2, F–H). In L1 animals subjected to miniSOG-mediated optogenetic ablation of transient electrical synapses, subsequent cold stimulation to drive TRPA-1–mediated calcium oscillations significantly reduced the proportion of PLM neurons exhibiting chemical synapse defects at L4, compared with ablated animals without cold stimulation (Fig. 2, J and K). This result demonstrates that restoring high-frequency calcium activity is sufficient to substantially rescue the chemical synapse defects caused by premature electrical synapse loss, indicating that electrical synapses promote chemical synaptogenesis at least in part through the calcium oscillations they generate.

### UNC-51 and UNC-76 function in the same genetic pathway to regulate electrical synapse elimination

**Figure 3.**
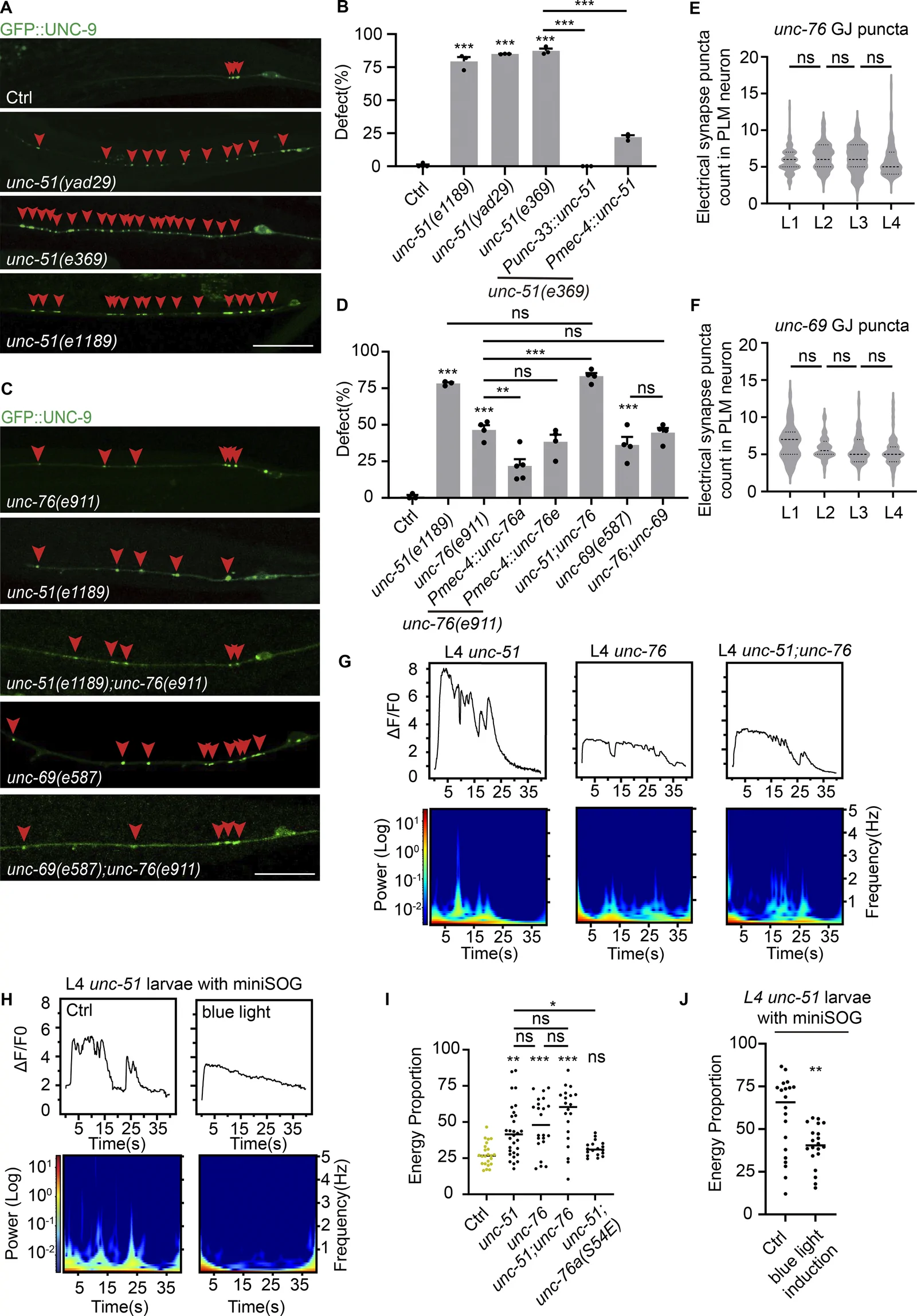
**UNC-51 and UNC-76 regulate electrical synapse elimination in mechanosensory neurons. (A)** Representative fluorescence images of GFP::UNC-9–labeled PLM electrical synapses in L4 control and *unc-51* mutant animals. Red arrowheads indicate labeled electrical synapses. **(B)** Quantification of excessive PLM electrical synapse defects in L4 control, *unc-51* mutant, and *unc-51* rescue animals. *n* >100 per genotype. **(C)** Representative fluorescence images of GFP::UNC-9–labeled PLM electrical synapses in L4 *unc-51*, *unc-76*, *unc-51*; *unc-76* double mutant, *unc-69*, and *unc-69*; *unc-76* double mutant animals. Red arrowheads indicate labeled electrical synapses. **(D)** Quantification of excessive PLM electrical synapse defects in L4 WT, *unc-51*, *unc-76*, *unc-76* rescue, *unc-51*; *unc-76* double mutant, *unc-69*, and *unc-69*; *unc-76* double mutant animals. *n* >100 per genotype. **(E)** Quantification of GFP::UNC-9–labeled electrical synapses in PLM neurons of *unc-76* mutant animals from L1 to L4 stages. *n* ≥ 65 per stage. **(F)** Quantification of GFP::UNC-9–labeled electrical synapses in PLM neurons of *unc-69* mutant animals from L1 to L4 stages. *n* ≥ 44 per stage. **(G)** Representative PLM calcium trace and wavelet spectrogram in L4 *unc-51*, *unc-76*, and *unc-51*; *unc-76* GCaMP6-expressing *C. elegans*. **(H)** Representative PLM calcium trace and wavelet spectrogram in L4 *unc-51; Pmec-4::miniSOG::unc-9* GCaMP6-expressing *C. elegans* with or without blue light activation. **(I)** Quantification of the secondary calcium signal energy proportion of PLM neurons in L4 *unc-51*, *unc-76*, *unc-51*; *unc-76* GCaMP6-expressing animals. *n* ≥18 per group. **(J)** Quantification of the secondary calcium signal energy proportion of PLM neurons in L4 *unc-51*; *Pmec-4::miniSOG::unc-9* GCaMP6-expressing *C. elegans* with or without blue light activation. *n* = 20 for each condition. Scale bars, 20 μm for confocal images, 1 μm for inset magnifications. Data are presented as the mean ± SEM. Statistical analyses were performed using one-way ANOVA with Tukey’s test (B, D, and F), Kruskal–Wallis test with Dunn’s test (E), Welch’s ANOVA with Dunnett’s T3 test (I), and Student’s *t* test with Welch’s correction (J). ns: not significant, *P < 0.05, **P < 0.01, ***P < 0.001.

**Figure S3.**
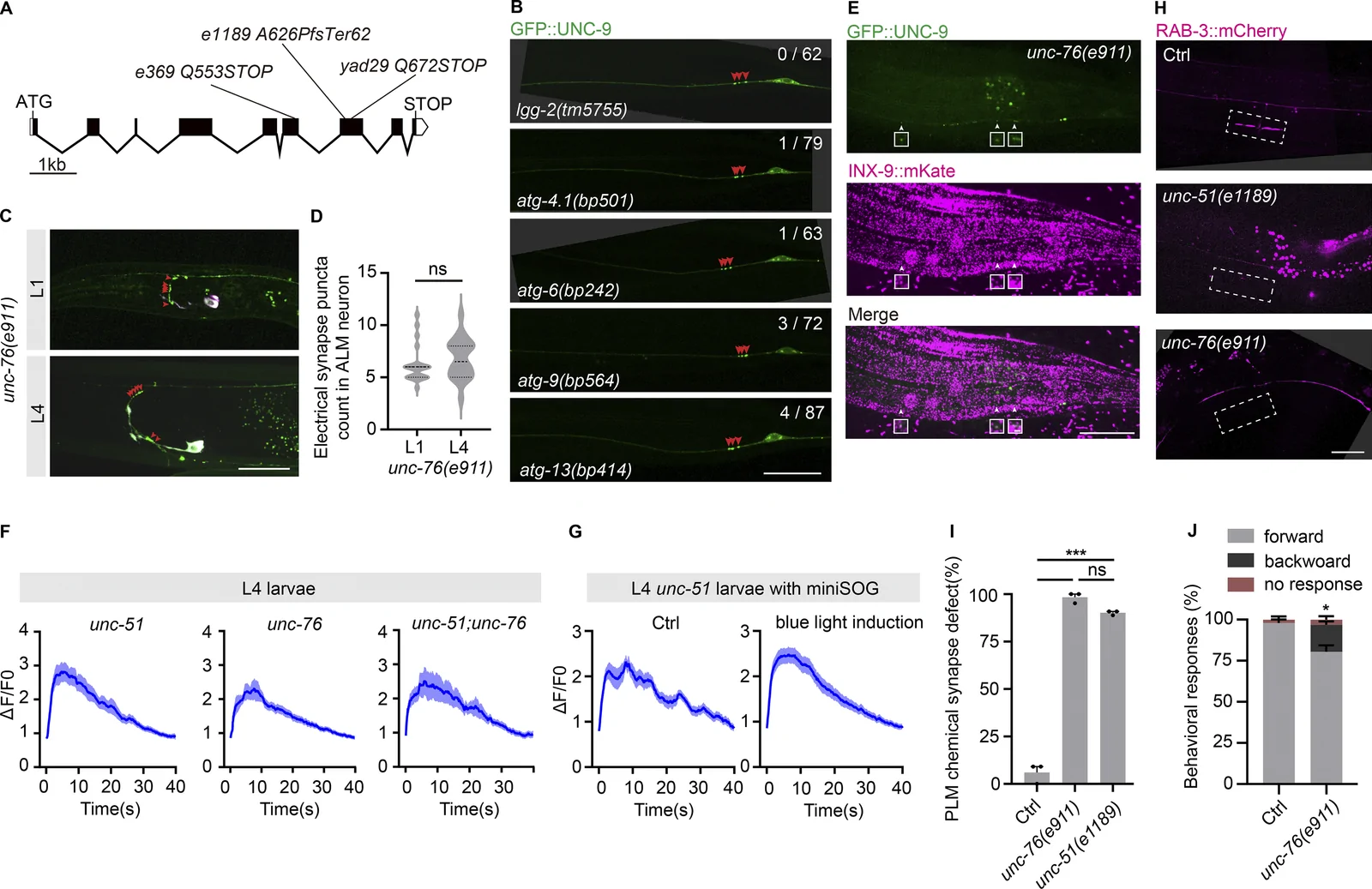
**Genetic characterization of the UNC-51/UNC-76 pathway. (A)** Schematic diagram illustrating different *unc-51* alleles with their respective mutation sites and mutation types. Scale bar: 1 kb. **(B)** Representative fluorescence images of GFP::UNC-9–labeled PLM electrical synapses in L4 *lgg-*2, *atg-4.1*, *atg-6*, *atg-9*, and *atg-13* animals. Numbers in the upper right corner indicate the number of animals exhibiting the excessive PLM electrical synapse defect phenotype over the total number of animals examined. **(C)** Representative fluorescence images of GFP::UNC-9–labeled electrical synapses in ALM neurons of *unc-76* mutant animals at L1 and L4 stages. **(D)** Quantification of GFP::UNC-9–labeled electrical synapses in ALM neurons of *unc-76* mutant animals at L1 and L4 stages. *n* = 20 for both stages. **(E)** Representative fluorescence images of colocalized epidermal INX-9 and PLM UNC-9 puncta in L4 *unc-76* animal (*Pdpy-7::inx-9::mKate; Pmec-4::GFP::unc-9*). White arrowheads point to colocalized electrical synapses. **(F)** Population-averaged calcium trace of PLM neurons in L4 *unc-51*, *unc-76*, *unc-51*; *unc-76* mutant animals. *n* = 30 (*unc-51*), 22 (*unc-76*), 20 (*unc-51*; *unc-76*). **(G)** Population-averaged calcium trace of PLM neurons in L4 *unc-51; Pmec-4::miniSOG::unc-9* GCaMP6-expressing *C. elegans* with or without blue light activation. *n* = 20 per condition. **(H and I)** Representative images of PLM chemical synaptic branches (H) and quantification of chemical synapse defects (I) in L4 Ctrl, *unc-51*, and *unc-76* animals. *n* = 38 (*Ctrl*), 49 (*unc-76*), 31 (*unc-51*). **(J)** Quantification of behavioral responses to gentle touch stimulation in L4-stage WT and *unc-76* animals. Forward movements were defined as normal responses, whereas backward movements and no response were classified as abnormal responses. The proportions of normal and abnormal responses were compared between genotypes. *n* > 100 per group. Scale bars, 20 μm. Data are presented as the mean ± SEM. Statistical analyses were performed using the Mann–Whitney test (D), one-way ANOVA with Tukey’s test (I), and Students’ *t* test (J). ns: not significant, *P < 0.05, ***P <0.001.

To uncover the regulatory mechanism underlying PLM electrical synapse elimination, we performed an unbiased ethyl methanesulfonate (EMS)–induced forward genetic screening using GFP::UNC-9 transgenic line (*yadIs12*) worms to identify genes that suppress electrical synapse elimination. Through the screening, we isolated the mutant *yad29*, in which PLM neurons exhibited abnormally excessive electrical synapses throughout the entire neurite at L4 (Fig. 3 A). Genetic mapping and rescue experiments identified *yad29* as a novel *unc-51* allele containing a single-nucleotide mutation introducing a premature stop codon at amino acid 672 (Fig. S3 A). Consistently, two independent *unc-51* alleles, *unc-51(e1189)* and *unc-51(e369)*, also exhibited similar excessive electrical synapses (Fig. 3 A). Quantification in L4 revealed that 79.3%, 85%, and 87.4% of L4 animals exhibited the defect in *unc-51(e1189)*, *unc-51(yad29)*, and *unc-51(e369)* strains, respectively—all significantly higher than WT (Fig. 3 B). Tissue-specific rescue experiments demonstrated that both pan-neuronal (*unc-33* promoter–driven) and PLM-specific (*mec-4* promoter–driven) UNC-51 expression significantly rescued the phenotype in *e369* worms, confirming a cell-autonomous requirement of UNC-51 for regulating PLM electrical synapse distribution (Fig. 3 B).

UNC-51/ULK is an evolutionarily conserved kinase best characterized for its role in initiating autophagy (Mizushima, 2010). To investigate how UNC-51 regulates electrical synapse development, we first accessed the involvement of autophagy by disrupting key autophagy-related genes (*atg-9*, *atg-6*, *atg-13*, *lgg-2*, *atg-4.1*). However, disruption of these autophagy genes did not reproduce the excessive electrical synapse phenotype (Fig. S3 B), demonstrating that UNC-51 regulates electrical synapses independently of its autophagy function.

Beyond autophagy, UNC-51/ULK has been implicated in vesicular trafficking and neuronal development (Ogura et al., 1994; Zhou et al., 2007; Toda et al., 2008). To identify downstream effectors of UNC-51 in electrical synapse elimination, we systematically screened candidate genes implicated in regulating neuronal development. Among the genes tested, *unc-76(e911)* mutation produced excessive electrical synapse phenotypes resembling *unc-51* mutants (Fig. 3 C). Quantification revealed that abnormal phenotypes were observed in 46.5% of *unc-76(e911)* mutants and 83.3% of *unc-76(e911)*; *unc-51(e1189)* double mutants, a frequency not significantly different from the 78.3% observed in *unc-51(e1189)* mutants (Fig. 3, C and D). These results indicate that UNC-51 and UNC-76 function within the same genetic pathway. Further screening of UNC-76–associated transport factors identified *unc-69* as an additional component of this pathway. *unc-69* encodes a coiled-coil protein homologous to mammalian SCOCO that directly interacts with UNC-76 to form kinesin-1 adaptor complexes mediating axonal transport (Su et al., 2006; Glomb et al., 2023). In *unc-69(e587)* mutants, excessive electrical synapses were observed in 36% of animals, and double mutant analysis of *unc-76(e911)*; *unc-69(e587)* revealed 44.6% affected animals, which was comparable to single mutants (Fig. 3, C and D), supporting that UNC-69 and UNC-76 function within the same regulatory pathway.

Collectively, these genetic analyses suggest that UNC-51 and UNC-76 act within the same genetic pathway governing electrical synapse distribution during neural development. To determine whether this pathway regulates developmental electrical synapse elimination, electrical synapse distribution across stages was examined in pathway mutants. Since *unc-51* mutants exhibit delayed mechanosensory neuron development at L1, we focused on *unc-76* mutants, which display relatively normal neurite development, to observe the elimination process. In PLM neurons of *unc-76* and *unc-69* mutants, the normal developmental reduction in electrical synapses was abolished across all four larval stages examined (Fig. 3, E and F). We note that *unc-76* mutants showed a modest reduction in gap junction number already at L1 relative to WT, likely reflecting a partial requirement for UNC-76 in anterograde innexin delivery during synapse assembly, distinct from its later, phosphorylation-dependent role in retrograde elimination. A similar failure to eliminate electrical synapses was observed in *unc-76* mutant ALM neurons (Fig. S3, C and D), indicating that this pathway is required for developmental electrical synapse elimination. Persistent UNC-9 puncta in unc-76 mutants continued to colocalize with hypodermal INX-9 at levels comparable to WT transient synapses (Fig. S3 E), indicating that these puncta remain at their native synaptic position rather than being redistributed to an ectopic site, and confirming that the *unc-76* phenotype reflects a failure of elimination rather than a general trafficking or positioning defect.

To investigate the functional consequences of impaired electrical synapse elimination, we performed calcium imaging in PLM neurons across multiple genotypes at L4 and quantified the secondary calcium signal energy proportion (as defined above). All mutants exhibited typical PLM calcium responses to mechanical stimulation, indicating that basic neuronal excitability was intact across genotypes (Fig. S3 F). However, both *unc-51* and *unc-76* single mutants exhibited significantly elevated energy proportion compared with WT controls (Fig. 3, G and I), suggesting that persistent electrical synapses drive abnormal high-frequency calcium dynamics. *unc-51*;*unc-76* double mutants displayed energy proportion levels comparable to either single mutant alone, consistent with UNC-51 and UNC-76 functioning within the same genetic pathway. To directly confirm that these aberrant oscillations originate from persistent transient gap junctions, we performed miniSOG-mediated optogenetic ablation of excess gap junctions in *unc-51* mutants, which significantly reduced the energy proportion (Fig. 3, H and J), providing direct causal evidence that the abnormal calcium dynamics in pathway mutants are driven by persistent transient electrical synapses that fail to be eliminated. Beyond their effects on calcium dynamics, both *unc-51*(90.2%) and *unc-76*(98.4%) mutants exhibited a significantly elevated proportion of PLM neurons with absent or severely abnormal chemical synapses at L4 (Fig. S3, H and I). We note, however, that these defects should be interpreted with some caution: UNC-51 also regulates autophagy at synapses required for normal presynaptic assembly (Stavoe et al., 2016; Dutta et al., 2023; Hassan and Hiesinger, 2023), and UNC-76 functions as a kinesin-1 cargo adaptor that may directly affect transport of chemical synapse components (Toda et al., 2008); the chemical synapse defects observed in these mutants therefore likely reflect multiple contributing mechanisms beyond electrical synapse dysregulation alone.

To examine the impact of these structural defects on functional consequences, we assessed gentle touch responses in *unc-76* mutants at L4—PLM’s chemical synapses onto the backward-command interneurons AVA and AVD have been proposed to inhibit the magnitude of reversals, thereby coordinating the directional switch to forward movement upon posterior touch (Chalfie et al., 1985). *unc-76* mutants exhibited a significantly higher proportion of touch response abnormalities (19.6%) compared with WT controls (2%) (Fig. S3 J). We did not extend this analysis to *unc-51* mutants: although *unc-51* mutants retain the ability to sense touch, their pronounced uncoordinated locomotion precludes reliable scoring of directional responses. Together, these results indicate that the chemical synapse defects arising from impaired electrical synapse elimination compromise mechanosensory circuit function.

### UNC-51 kinase controls electrical synapse elimination via UNC-76 phosphorylation

**Figure S4.**
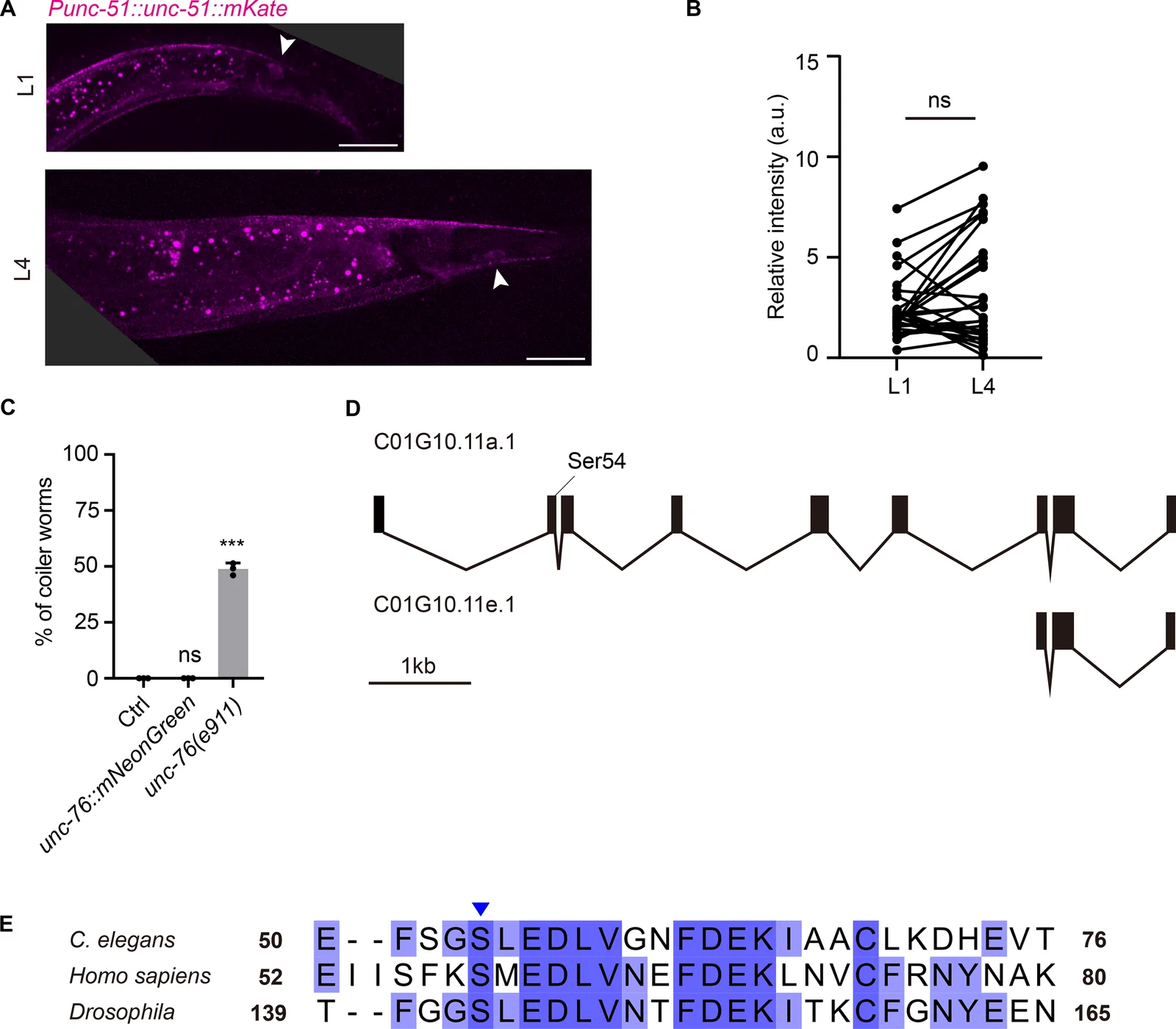
**UNC-51 expression, UNC-76::mNeonGreen knock-in validation, and Ser54 site identification. (A)** Representative fluorescence images showing UNC-51 expression in the tail region of L1 and L4 *Punc-51::unc-51::mKate* worms. Red arrowheads indicate PLM cell bodies. **(B)** Quantification of changes in PLM cell body fluorescence intensity in individual *Punc-51::unc-51::mKate* animals tracked from L1 to L4 stages. *n* = 28 per stage. **(C)** Quantification of coiler locomotion defects in WT, *unc-76::mNeonGreen* knock-in, and *unc-76* mutant animals. *n* = 506 (WT), *n* = 449 (*unc-76::mNeonGreen*), *n* = 571 (*unc-76(e911)*). **(D)** Schematic diagram illustrating UNC-76 long isoform a and short isoform e, with the conserved serine 54 phosphorylation site indicated. Scale bar: 1 kb. **(E)** Sequence comparison of UNC-76/FEZ1 from *Drosophila*, *C. elegans*, and human, highlighting conserved regions surrounding the putative UNC-51 phosphorylation serine residue. The blue arrow points to the putative phosphorylation serine residue. Error bars represent the mean ± SEM. Statistical analyses were performed using a paired *t* test (B) and one-way ANOVA with Tukey’s test (C). ns: not significant, ***P < 0.001.

**Figure 4.**
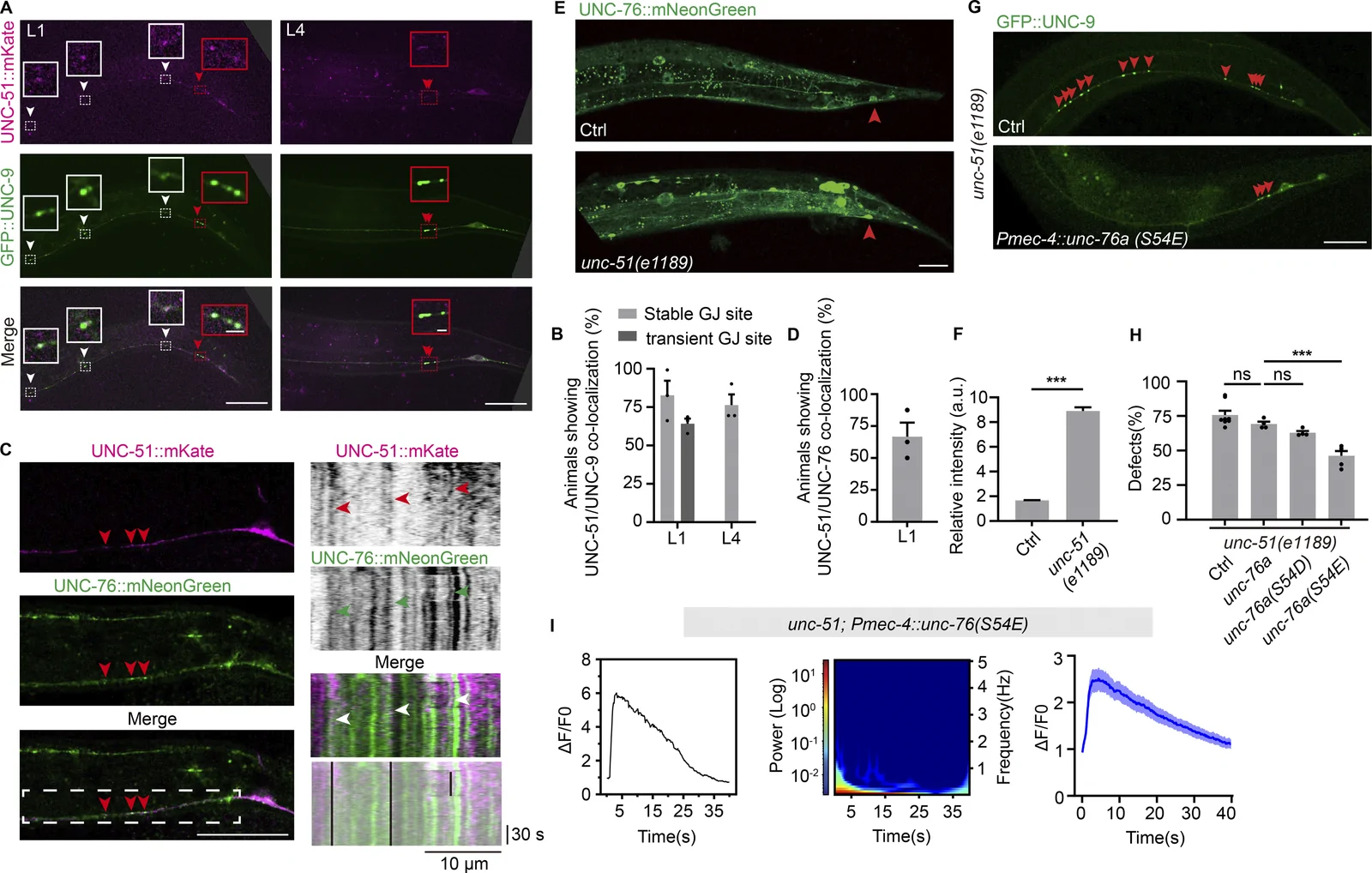
**UNC-51 kinase controls electrical synapse elimination via UNC-76 phosphorylation. (A)** Representative fluorescence images of GFP::UNC-9 and UNC-51::mKate puncta along the PLM axon in L1 and L4 worms. Red and white arrowheads indicate colocalized stable and transient electrical synapses, respectively. **(B)** Quantification of the percentage of worms exhibiting colocalized UNC-51::mKate and GFP::UNC-9 puncta at electrical synapse sites. In L1 worms, colocalization was quantified separately at transient and stable electrical synapse sites, whereas in L4 worms, colocalization was assessed at stable electrical synapse sites. *n* > 30 per stage. **(C)** Representative fluorescence images and kymograph of UNC-76::mNeonGreen and UNC-51::mKate puncta along the PLM axon in L1 and L4 worms. For fluorescence image, red arrowheads point to colocalized puncta, while the rectangular box delineates the PLM axonal region used for time-lapse analysis. For kymograph, arrows and black lines indicate the positions of colocalized puncta. **(D)** Quantification of the percentage of L1 worms exhibiting colocalized UNC-51::mKate and UNC-76::mNeonGreen puncta along PLM axon. *n* = 24. **(E and F)** Representative fluorescence images (E) and quantification (F) of endogenous UNC-76::mNeonGreen in PLM neurons of L4 control and *unc-51* mutant animals. Red arrowheads in E indicate PLM neuron cell bodies. *n* = 102 (Ctrl), *n* = 105 (*unc-51*). **(G)** Representative fluorescence images of GFP::UNC-9–labeled PLM electrical synapses in L4 *unc-51* mutant animals and *unc-51* mutant animals expressing the UNC-76(S54E) phosphomimetic mutants. Red arrowheads point to labeled electrical synapses. **(H)** Quantification of excessive PLM electrical synapse defects in *unc-51* mutant animals and *unc-51* worms expressing UNC-76 isoform a, UNC-76(S54D) phosphomimetic, or UNC-76(S54E) phosphomimetic mutants. *n* > 100 per genotype. **(I)** Representative calcium trace, wavelet spectrogram, and population-averaged calcium trace of PLM neurons in *L4 unc-51*; *Pmec-4::unc-76(S54E)* GCaMP6-expressing *C. elegans*. *n* = 18. Scale bars, 20 μm for confocal images, 2 μm for inset magnifications, and 10 μm (horizontal) and 30 s (vertical) for time-lapse images; data are presented as the mean ± SEM. Statistical analyses were performed using the Mann–Whitney test (F), and one-way ANOVA with Tukey’s test (H). ns: not significant, ***P <0.001.

Having identified the genetic pathway, we next investigated the molecular basis of UNC-51 function, first examining its expression levels and subcellular localization at L1 and L4 to probe the developmental specificity of its role in electrical synapse elimination. Using a *Punc-51::unc-51::mKate* reporter strain, we found no significant difference in UNC-51 tissue distribution or fluorescence intensity in PLM cell bodies between L1 and L4 stages (Fig. S4, A and B). To examine UNC-51 subcellular localization specifically within PLM neurons, we generated *Pmec-4::unc-51::mKate* transgenic animals. UNC-51 colocalized with both transient and stable gap junctions along PLM axons at L1, and was primarily associated with stable gap junctions at L4 (Fig. 4, A and B), consistent with a shift in UNC-51 localization rather than expression.

We next examined the relative subcellular localization of UNC-51 and UNC-76. To this end, the C terminus of UNC-76 was endogenously tagged with mNeonGreen using CRISPR-mediated knock-in. The *unc-76::mNeonGreen* knock-in strain retained normal function, as evidenced by the absence of coiler phenotypes, with defect levels comparable to WT and significantly lower than those observed in *unc-76* mutants, in which 48.8% exhibited the defect (Fig. S4 C). We then crossed the *Pmec-4::unc-51::mKate* transgene to the endogenous *unc-76::mNeonGreen* background. In L1 PLM neurons, UNC-51::mKate and UNC-76::mNeonGreen colocalized along the neurite, both in static images and throughout time-lapse imaging (Fig. 4 C), indicating that UNC-51 and UNC-76 are present at the same subcellular sites where transient electrical synapses are being eliminated.

Given that ULK-family kinases have been implicated in regulating UNC-76/FEZ1 function in other systems (Toda et al., 2008), we asked whether UNC-51 similarly functions upstream of UNC-76 in *C. elegans*. In *unc-51(e1189)* mutants, UNC-76::mNeonGreen levels were approximately fivefold higher than in WT neurons (Fig. 4, E and F), indicating that UNC-51 negatively regulates UNC-76 protein abundance, and functions upstream of UNC-76 in the regulatory pathway.

Having established that UNC-51 and UNC-76 colocalize and that UNC-51 negatively regulates UNC-76 abundance, we next asked whether this regulation reflects a direct biochemical interaction. Previous studies in *Drosophila* demonstrated that UNC-51 phosphorylates UNC-76 at Ser143 to regulate axonal transport by mediating motor–cargo assembly (Toda et al., 2008). A conserved homologous phosphorylation site, Ser54, was identified in *C. elegans* UNC-76 long isoform (isoform a) (Fig. S4, D and E). To determine whether UNC-51 regulates UNC-76 through phosphorylation, phosphomimetic mutants UNC-76(S54D) and UNC-76(S54E) were constructed, in which Ser54 was substituted with Asp or Glu to mimic constitutive phosphorylation. Remarkably, the expression of UNC-76(S54E) in *unc-51(e1189)* mutant worms partially rescued the excessive electrical synapse phenotypes, whereas WT UNC-76 expression showed no rescue effect (Fig. 4, G and H). These results demonstrate that phosphorylation at Ser54 is both necessary and sufficient for UNC-76 function in electrical synapse elimination, suggesting that UNC-51 kinase directly phosphorylates UNC-76 to regulate electrical synapse elimination process.

To further validate the functional significance of UNC-51–mediated UNC-76 phosphorylation, we examined whether the expression of phosphomimetic UNC-76(S54E) could rescue the aberrant calcium oscillations observed in *unc-51* mutants at L4. The expression of UNC-76(S54E) in *unc-51* mutants significantly suppressed the elevated energy proportion toward WT levels (Fig. 4 I and Fig. 3 I), directly demonstrating that phosphorylation of UNC-76 at Ser54 is required not only for electrical synapse elimination but also for the consequent termination of high-frequency calcium dynamics. Together, these functional data establish that the UNC-51→UNC-76 phosphorylation axis drives electrical synapse elimination and the precise developmental termination of transient electrical synapse–driven calcium oscillations.

### UNC-76 phosphorylation promotes retrograde removal of innexins from electrical synapses

**Figure 5.**
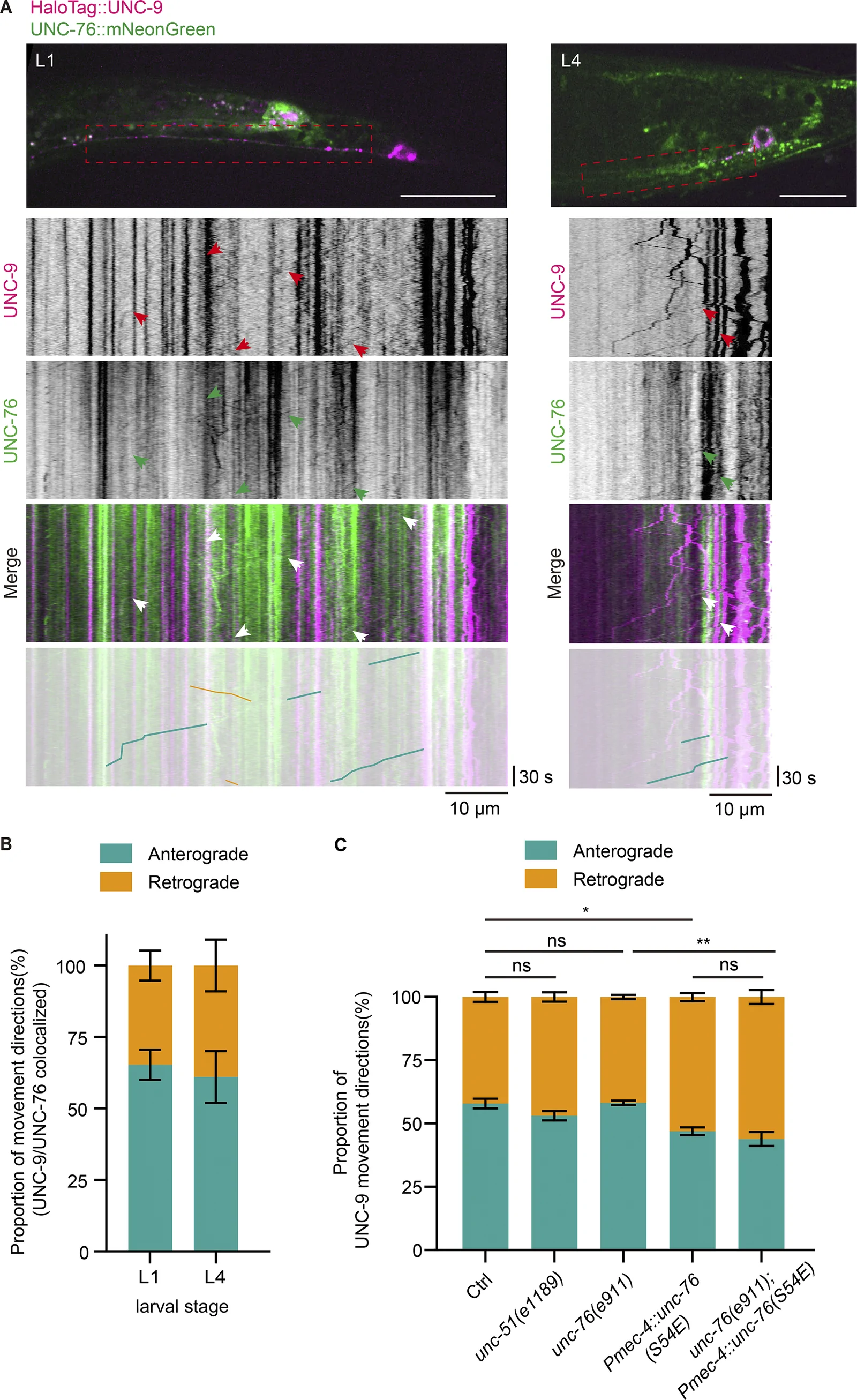
**UNC-76 colocalizes with UNC-9 and exhibits stage-specific directional transport. (A)** Representative fluorescence and time-lapse images of electrical synapse dynamics visualized using HaloTag::UNC-9 and endogenous UNC-76::mNeonGreen in L1 and L4 animals. The red rectangular box delineates the PLM axonal region used for time-lapse analysis. Arrowheads point to colocalized UNC-76 and UNC-9 punctum movement. The blue and yellow lines indicate UNC-9 anterograde and retrograde movement, respectively. **(B)** Quantification of the directional movement of colocalized UNC-76 and UNC-9 puncta in anterograde and retrograde directions at L1 and L4. *n* = 21 (L1), *n* = 13 (L4). **(C)** Quantification of anterograde and retrograde PLM UNC-9 movements in L1 control, *unc-51*, *unc-76*, *pmec-4::unc-76(S54E)*, and *unc-76*; *pmec-4::unc-76(S54E)* animals. *n* ≥ 15 per genotype. Scale bars, 20 μm for confocal images, and 10 μm (horizontal) and 30 s (vertical) for time-lapse images; data are presented as the mean ± SEM. Statistical analyses were performed using one-way ANOVA with Tukey’s test. ns: not significant, *P < 0.05, **P < 0.01.

**Figure S5.**
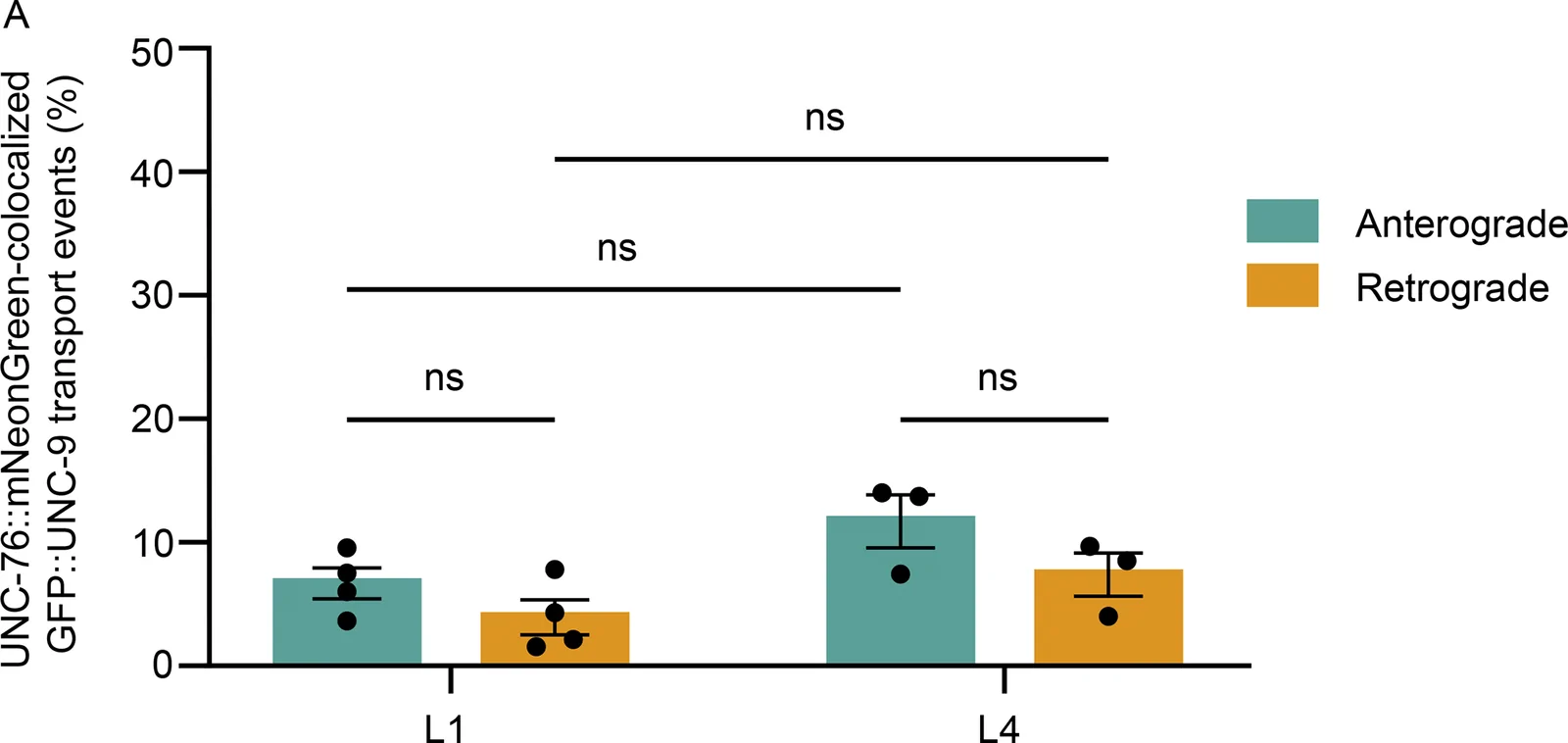
**Quantification of UNC-9–UNC-76 co-transport events.** **(A)** Quantification of the proportion of GFP::UNC-9 transport events cotransported with UNC-76::mNeonGreen among all GFP::UNC-9 transport events during anterograde and retrograde movement in L1 and L4 animals. *n* = 21 (L1), *n* = 13 (L4). Data are presented as the mean ± SEM. Statistical analyses were performed using two-way ANOVA with Tukey’s test. ns: not significant.

To determine the cellular basis of this phosphorylation-dependent regulation, we generated *Pmec-4::HaloTag::UNC-9*;*UNC-76::mNeonGreen* dual-labeled strains to simultaneously visualize both proteins. Time-lapse imaging of PLM axons revealed cotransport of UNC-9 and UNC-76 on moving vesicles in both anterograde (soma to axon) and retrograde (axon to soma) directions at L1 and L4 (Fig. 5 A). Quantitative analysis revealed that transport patterns were consistent across developmental stages. In L1, anterograde cotransport occurred in 65% of cases, while L4 animals showed that in 61% of cases, with no significant difference between stages (Fig. 5 B). These findings demonstrate that UNC-76 and UNC-9 are cotransported bidirectionally along PLM axons; across the total pool of UNC-9 transport events, however, only a small fraction (∼7%, Fig. S5 A) showed detectable UNC-76 cotransport, consistent with dilution by the high rate of constitutive gap junction turnover that dominates overall UNC-9 trafficking.

We next asked whether UNC-51 and UNC-76 influence innexin transport and removal from electrical synaptic sites. We first assessed UNC-9 movements in *unc-76* loss-of-function mutants. We found that neither *unc-51* nor *unc-76* mutants displayed significant alteration in the ratio of retrograde to anterograde UNC-9 movements compared with L1 controls (Fig. 5 C), indicating that loss of either the kinase (UNC-51) or its substrate (UNC-76) alone is not sufficient to alter the balance of UNC-9 transport. We next expressed phosphomimetic UNC-76 (S54E) in both WT and *unc-76* mutant backgrounds. The expression of phosphomimetic UNC-76 significantly increased the proportion of retrograde UNC-9 movements both in WT animals and in *unc-76* mutants (Fig. 5 C). The consistent gain-of-function effect across both backgrounds demonstrates that phosphorylated UNC-76 is sufficient to promote removal of UNC-9 from the electrical synaptic site. Together, these results indicate that UNC-76 cotraffics with innexins along PLM axons and that phosphorylation of UNC-76 at Ser54 is sufficient to promote net retrograde removal of innexins from electrical synapses.

### RAB-10 cooperates with UNC-76 to drive electrical synapse elimination

**Figure 6.**
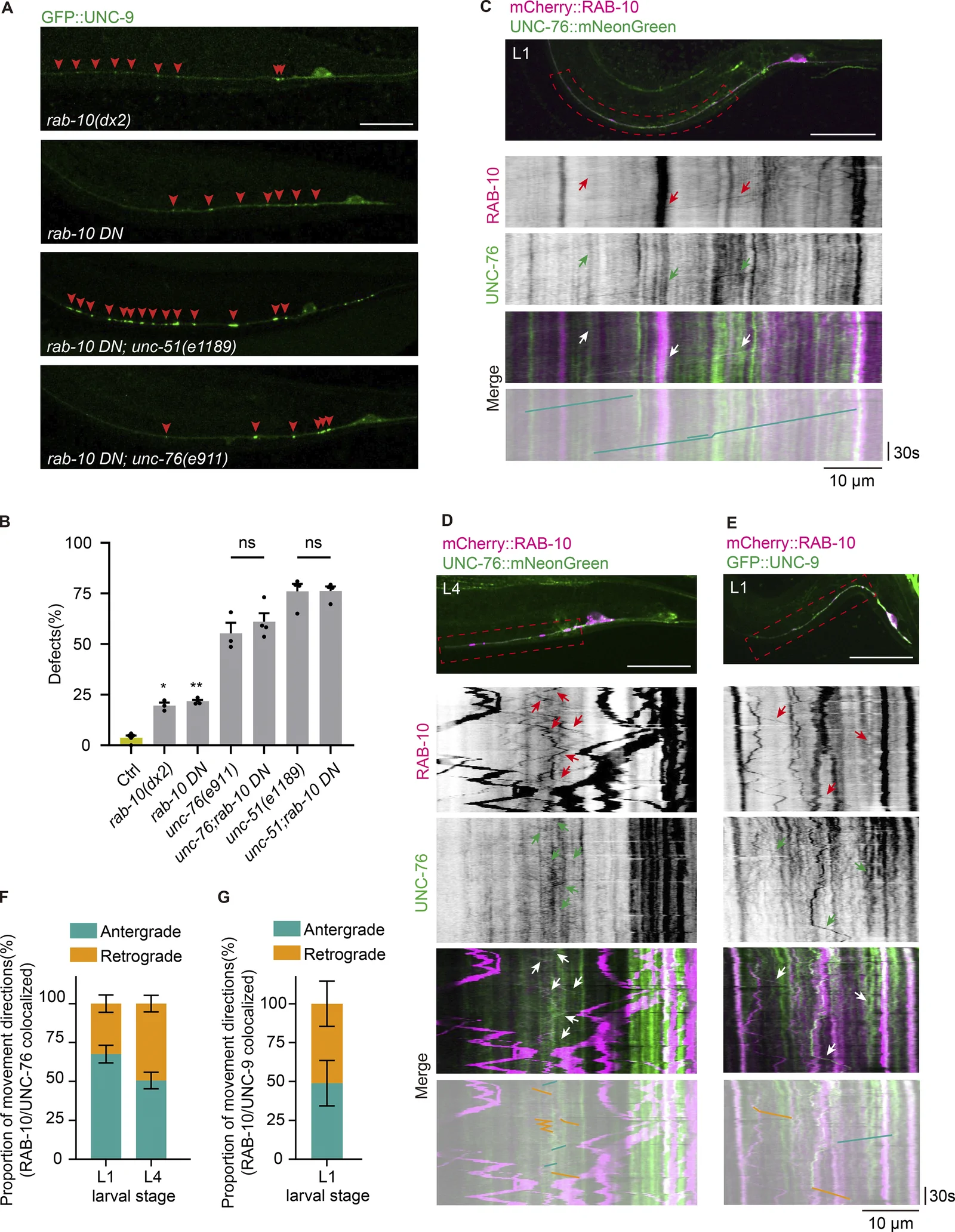
**RAB-10 functions with UNC-76 to regulate electrical synapse elimination. (A and B)** Representative fluorescence images (A) and quantification (B) of GFP::UNC-9–labeled PLM electrical synapses in L4 *rab-10(dx2)*, *rab-10(DN)*, *rab-10(DN)*; *unc-51(e1189)*, and *rab-10(DN)*; *unc-76(e911)* animals. Red arrowheads in A indicate labeled electrical synapses. *n* > 80 per genotype. **(C and D)** Representative fluorescence and time-lapse images showing colocalization of mCherry::RAB-10 and UNC-76::mNeonGreen punctum movement in PLM neurons at L1 (C) and L4 (D). Arrowheads point to colocalized punctum movements. The blue and yellow lines indicate anterograde and retrograde movement, respectively. **(E)** Representative fluorescence and time-lapse images showing colocalization of mCherry::RAB-10 and GFP::UNC-9 in PLM neurons at the L1 stage. **(F)** Quantification of the colocalized UNC-76 and RAB-10 punctum directional movement in anterograde and retrograde directions at L1 and L4. *n* = 16 (L1), *n* = 12 (L4). **(G)** Quantification of the colocalized RAB-10 and UNC-9 punctum directional movement in anterograde and retrograde directions at L1. *n* = 11. Scale bars, 20 μm for confocal images, and 10 μm (horizontal) and 30 s (vertical) for time-lapse images; data are presented as the mean ± SEM. Statistical analyses were performed using one-way ANOVA with Tukey’s test (B). ns: not significant, *P < 0.05, **P < 0.01.

**Figure S6.**
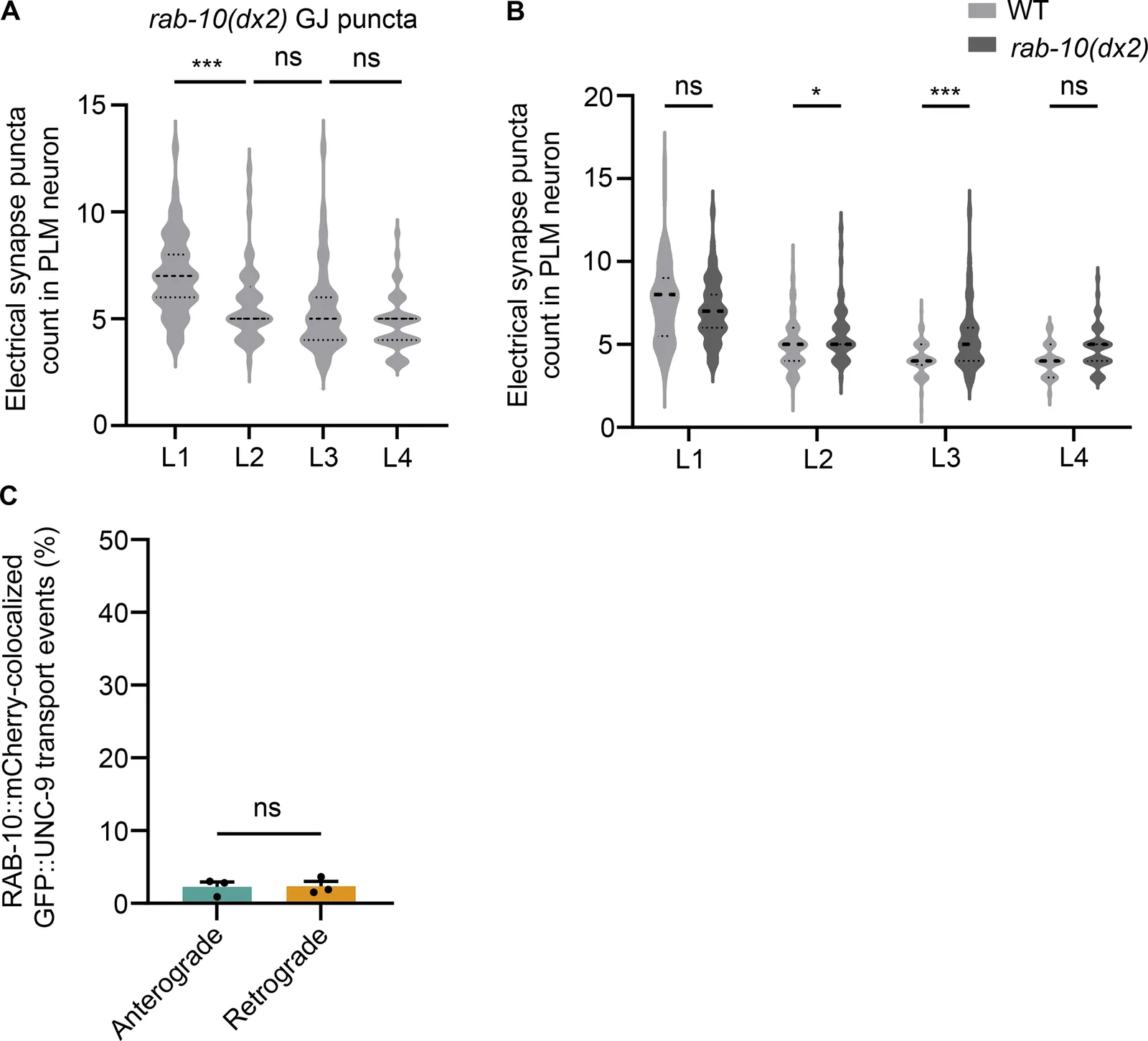
***rab-10* mutant phenotype and UNC-9–RAB-10 co-transport quantification. (A)** Quantification of GFP::UNC-9–labeled electrical synapses in PLM neurons of *rab-10* mutant animals at each larval stage (L1–L4). *n* ≥ 48 per stage. **(B)** Comparison of PLM electrical synapse numbers between WT and *rab-10* mutant animals at each larval stage (L1–L4). *n* ≥ 48 per stage. **(C)** Quantification of the proportion of GFP::UNC-9 transport events cotransported with RAB-10::mCherry among all GFP::UNC-9 transport events during anterograde and retrograde transport in L1 animals. *n* = 11 for both stages. Data are presented as the mean ± SEM. Statistical analyses were performed using one-way (A) and two-way (B) ANOVA with Tukey’s test, and Student’s *t* test (C). ns: not significant, *P < 0.05, ***P < 0.001.

Having established that electrical synapse elimination requires active transport, the vesicular machinery responsible for this process was next investigated. Systematic screening of transport-related genes revealed that disruption of RAB-10 function by either *rab-10(dx2)* mutants or expression of GDP-locked, dominant-negative RAB-10(T23N) reproduced electrical synapse elimination defects (Fig. 6, A and B). RAB-10 is a small GTPase known to regulate endosomal trafficking and has been implicated in receptor trafficking and degradation in both *C. elegans* and mammalian systems (Glodowski et al., 2007; Stenmark, 2009). To determine whether RAB-10 functions in the same genetic pathway as UNC-51 and UNC-76, genetic interaction analysis was performed. Because *unc-51(e1189)*;*rab-10(dx2)* and *unc-76(e911)*;*rab-10(dx2)* double mutants exhibited severe sterility that precludes phenotypic analysis, we instead expressed a dominant-negative RAB-10(T23N) transgene in PLM neurons of *unc-51(e1189)* or *unc-76(e911)* mutant backgrounds as an alternative approach. The overexpression of RAB-10(T23N) in *unc-51* or *unc-76* mutants did not exacerbate the electrical synapse defects observed in the respective single mutants (Fig. 6, A and B). This lack of additive effects suggests that RAB-10 functions within the same genetic pathway as UNC-51 and UNC-76. Consistent with this, developmental stage analysis revealed that *rab-10* mutants also failed to eliminate electrical synapses, with gap junction numbers remaining largely unchanged from L1 to L4 (Fig. S6, A and B), further supporting a shared role for RAB-10 in the developmental elimination process.

To determine whether RAB-10 works cooperatively with UNC-76, cotrafficking analysis using mCherry::RAB-10 in *unc-76::mNeonGreen* animal PLM neurons was performed. Time-lapse imaging at L1 and L4 revealed extensive cotrafficking of UNC-76 and RAB-10–positive vesicles in both anterograde and retrograde directions, with 68% anterograde movements in L1 and 51% in L4 (Fig. 6, C, D, and F), indicating that UNC-76 directly associates with RAB-10–positive vesicles in axonal transport. Additionally, colocalized axonal transport of RAB-10–positive vesicles with UNC-9 was also observed, with 49% of movements occurring in the anterograde direction (Fig. 6, E and G), demonstrating a functional link between RAB-10–positive vesicle trafficking and innexin transport as with the UNC-76 cotransport data above, only a small fraction of total UNC-9 movements (∼2%, Fig. S6 C) showed detectable RAB-10 cotransport, reflecting the same dilution by constitutive gap junction turnover.

Collectively, these findings establish RAB-10–positive vesicles as the key transport machinery that cooperates with UNC-76 to mediate active removal of innexin components during developmental electrical synapse elimination.

## Discussion

**Figure 7.**
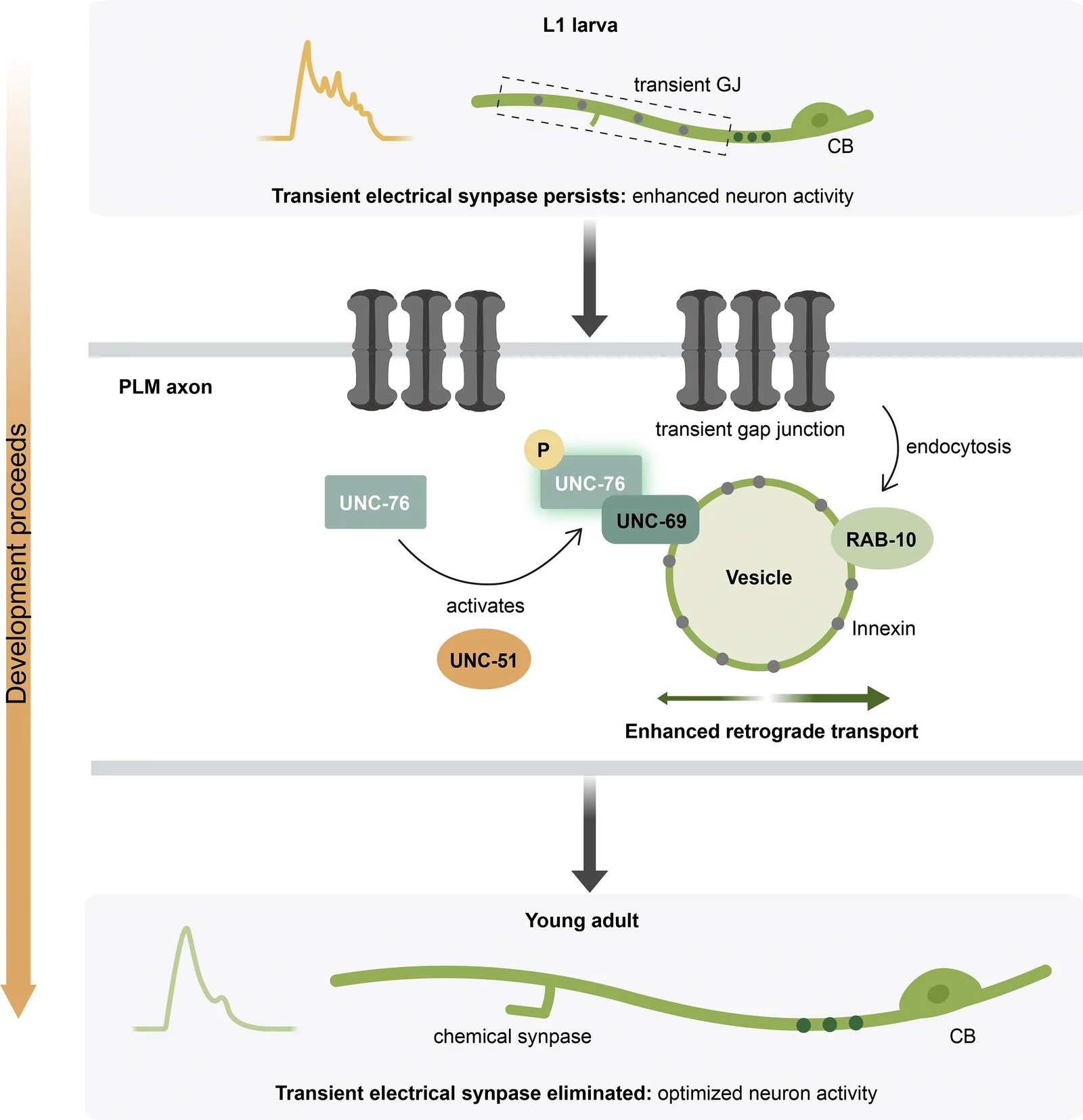
Schematic model illustrating the UNC-51–UNC-76–RAB-10 signaling pathway that regulates transient electrical synapse elimination, which is essential for normal neuronal activity and chemical synapse development.

Widespread electrical coupling between developing neurons and their coordinated synchronous activity have been observed across systems, from the zebrafish spinal cord to the mammalian cortex (Saint-Amant and Drapeau, 2001; Yu et al., 2012). However, how these transient connections are precisely terminated and the molecular mechanisms governing their elimination have remained unknown. Here, we identify the UNC-51/UNC-76 pathway as the core machinery for programmed electrical synapse elimination and demonstrate its functional necessity for proper circuit maturation (Fig. 7).

### A phosphorylation switch converts constitutive trafficking into programmed elimination

Our findings reveal how developmental elimination is distinguished from constitutive turnover through temporal regulation of trafficking machinery. We propose that UNC-51–mediated phosphorylation of UNC-76 functions as a molecular switch that converts balanced bidirectional trafficking into directional removal (Fig. 7).

At early larval stages, electrical synapses in dynamic equilibrium are maintained by balanced trafficking. UNC-76 and RAB-10 continuously mediate both delivery and retrieval of innexins, resulting in stable synapse numbers despite rapid protein exchange. Loss of UNC-76 alone does not alter the retrograde-to-anterograde ratio of UNC-9 movements, consistent with a model in which UNC-76 acts specifically at the regulated, phosphorylation-dependent step of elimination rather than in maintaining the constitutive baseline balance of transport. We propose that this regulatory transition is triggered when UNC-51 kinase phosphorylates UNC-76 at Ser54, RAB-10 continuously internalizes UNC-9 from the synaptic membrane as part of this constitutive cycle, and phosphorylated UNC-76 acts on the resulting RAB-10–positive endosomes to bias their transport toward the retrograde direction, converting balanced flux into net protein removal—a model consistent with the established roles of RAB-10 in endosomal trafficking and UNC-76 as a kinesin-1 cargo adaptor. Formally distinguishing UNC-9 internalization from its subsequent retrograde transport remains technically challenging in the fine axonal processes of PLM neurons, and we cannot exclude that phosphorylated UNC-76 also promotes RAB-10–mediated internalization itself rather than acting solely at the transport step; resolving the precise division of labor between these two steps is an important goal for future work. The economy of this mechanism is notable: the same molecular components execute both constitutive turnover and developmental elimination, with UNC-76 phosphorylation state determining the outcome. The low fraction of UNC-9 movements involving UNC-76 cotransport reflects the dilution of elimination-specific events by constitutive gap junction maintenance, which occurs continuously and at high turnover rates for both transient and stable junctions in PLM neurons. Retrieved innexins enter RAB-10–positive compartments, but whether these preferentially traffic to degradative versus recycling pathways during elimination is unclear. This model explains why disrupting any pathway component produces similar phenotypes and why rapid turnover coexists with stable synapse numbers at L1. The developmental restriction of this switch to L1 does not reflect a change in UNC-51 abundance, which is comparable between L1 and L4 (Fig. S4, A and B), but rather a shift in its subcellular localization: UNC-51 associates with both transient and stable gap junctions at L1 but becomes restricted to stable junctions by L4 (Fig. 4 A), suggesting that spatial enrichment at transient junctions—rather than expression timing—confers stage-specific elimination competence. It remains unclear, however, why elimination is developmentally restricted to L1, or what regulates UNC-51 localization or activity at transient junctions during this window. Notably, each larval molt is accompanied by a genome-wide oscillatory transcriptional program in which thousands of genes cycle in phase with the molting cycle (Hendriks et al., 2014)—including core timing regulators such as *lin-42*/PERIOD and *nhr-23*, as well as structural genes such as cuticle collagens (Frand et al., 2005)—raising the possibility that UNC-51, or an upstream regulator of its localization, lies downstream of this oscillator.

The architectural simplicity of electrical synapses—consisting primarily of clustered gap junction channels—makes cargo-selective transport particularly efficient. Unlike chemical synapse elimination, which requires multipathway mechanisms acting on diverse synaptic components, coordinated retrieval of the principal structural component is sufficient to dismantle the entire functional unit. For such architecturally streamlined synapses, cargo-selective vesicular trafficking thus offers an economical solution for developmental elimination.

### Temporal regulation of electrical synapse elimination is required for sequential circuit maturation

Transient electrical synapses in PLM neurons generate high-frequency calcium oscillations during early larval development, and their loss concurrently abolishes these dynamics and impairs chemical synapse formation. Using a loss-of-function/gain-of-function optogenetic strategy, we show that this relationship is causal rather than merely correlative at each step. Acute, stage-specific ablation of transient electrical synapses was sufficient to abolish high-frequency calcium oscillations without altering basal PLM excitability, demonstrating that these junctions actively drive—rather than simply accompany—the oscillatory activity of L1 neurons. This loss of oscillatory activity was in turn accompanied by a marked increase in chemical synapse defects. Critically, restoring high-frequency calcium activity independently of gap junction coupling, via TRPA-1–mediated cold stimulation after electrical synapse ablation, substantially rescued these chemical synapse defects. Together, these three manipulations—ablating electrical synapses, observing the resulting loss of both oscillations and chemical synapses, and then restoring oscillations to rescue synaptogenesis—establish a causal chain in which transient electrical synapses promote chemical synapse formation largely through the high-frequency calcium oscillations they generate. The rescue was partial rather than complete, however, indicating that calcium oscillations are not the sole instructive signal downstream of electrical coupling, and that electrical synapses likely contribute to chemical synaptogenesis through additional, calcium-independent mechanisms as well. Conversely, when elimination fails, persistent electrical synapses sustain aberrant high-frequency calcium oscillations well into later larval stages, and this is likewise accompanied by defective chemical synapse formation—demonstrating that timely elimination is equally required to terminate early network activity and permit functional specialization. Together, these bidirectional perturbations indicate that proper circuit maturation depends not simply on the presence or absence of transient electrical synapse activity, but on its precise temporal confinement to the appropriate developmental window: activity that is insufficient, activity that persists too long, or activity that occurs at the wrong stage can each disrupt chemical synapse maturation. This temporally bounded requirement for network activity is well established in other developmental contexts, including spontaneous retinal waves that must be precisely terminated to permit experience-dependent refinement (Blankenship and Feller, 2010), the elimination of polyneuronal innervation at the neuromuscular junction (Sanes and Lichtman, 1999), and the opening and closing of cortical critical periods, where either premature closure or prolonged extension impairs circuit maturation (Hensch, 2005).

The identity of the transient gap junction partners remains to be definitively established. Our colocalization analysis with the hypodermal innexin INX-9 suggests that hypodermal cells may represent one class of transient partners, though functional validation has been technically challenging due to the absence of detectable calcium dynamics in hypodermal cells under our imaging conditions. Whether these transient connections represent neuron–hypodermal gap junctions or involve other unidentified partners remains an open question for future investigation.

### Evolutionary conservation of the UNC-51/UNC-76 pathway

Our mechanistic analysis reveals that electrical synapse elimination requires active retrograde transport via the UNC-51→UNC-76→RAB-10 pathway. This pathway selectively removes innexins while presumably sparing other membrane components, indicating cargo specificity in the elimination machinery. UNC-51, the *C. elegans* homolog of mammalian ULK1/2, is best known for its role in autophagy initiation. However, our findings demonstrate an autophagy-independent function in regulating vesicular trafficking, consistent with emerging evidence that UNC-51/ULK proteins have diverse cellular roles beyond autophagy (Chan et al., 2007; Toda et al., 2008). The epistatic relationship between UNC-51 and UNC-76—reflected in the comparable phenotypes of *unc-51*;*unc-76* double mutants and either single mutant alone, and in the partial rescue of *unc-51* defects by phosphomimetic UNC-76(S54E)—places these two proteins within a common genetic pathway governing electrical synapse elimination. The approximately fivefold elevation of UNC-76 protein levels observed in *unc-51* mutants (Fig. 4, E and F) may reflect a compensatory upregulation triggered by loss of the phosphorylated, active form of UNC-76; however, since increased UNC-76 abundance alone did not rescue the *unc-51* phenotype, it is the phosphorylated state of UNC-76, rather than its overall abundance, that appears critical for electrical synapse elimination, and the basis for this apparent upregulation remains to be determined. Although this phosphorylation-dependent relationship is supported by genetic epistasis and by the rescuing activity of phosphomimetic UNC-76(S54E), we were unable to directly detect phosphorylated UNC-76 in vivo using an antibody-independent Phos-tag–based assay (Kinoshita et al., 2006), likely reflecting the low abundance of the phosphorylated species; the dynamic colocalization of UNC-51 with UNC-76 (Fig. 4 C) and with gap junctions along the PLM axon (Fig. 4 A) therefore provides complementary, albeit indirect, support for this interaction. However, the stronger phenotype of *unc-51* mutants relative to *unc-76* mutants, the incomplete epistasis between the two, and the partial rescue by UNC-76(S54E) together suggest that additional effectors downstream of UNC-51 likely contribute to electrical synapse elimination. Given that UNC-51/ULK1 directly phosphorylates multiple substrates across diverse cellular processes (Di Bartolomeo et al., 2010; Konno et al., 2013; Papinski et al., 2014), it is possible that additional UNC-51 substrates remain to be identified in this context. The net reduction in gap junctions suggests preferential removal, though the precise fate of retrieved innexins remains to be determined. The UNC-51 phosphorylation site on UNC-76/FEZ is evolutionarily conserved from invertebrates to mammals. Together with the established roles of ULK and FEZ proteins in mammalian axonal transport and synaptic development, this suggests that similar mechanisms may regulate connexin trafficking in vertebrate electrical synapses. Connexin-based electrical synapses in the mammalian brain also undergo developmental elimination in multiple regions, though the molecular pathways governing this process remain largely unknown. It will be important to determine whether ULK-mediated phosphorylation of FEZ proteins similarly regulates connexin trafficking in vertebrate neurons, which would indicate evolutionary conservation of this elimination mechanism from invertebrates to vertebrates.

### Broader implications for circuit refinement

Our work establishes cargo-selective retrograde transport as the mechanism for developmental electrical synapse elimination and provides a molecular explanation for how transient synaptic architectures are precisely removed after completing their developmental functions. The finding that UNC-51 phosphorylation converts constitutive trafficking into active removal reveals a general principle: developmental transitions can be achieved by temporally regulating the activity state of constitutive cellular machinery. Given the evolutionary conservation of the pathway components and the widespread occurrence of electrical synapse elimination during mammalian brain development, these mechanisms likely represent fundamental principles of neural circuit assembly that extend beyond *C. elegans*.

## Materials and methods

### *C. elegans* strains and genetics

*C. elegans* strains were maintained on NGM plates seeded with OP50 *Escherichia coli* at 20–23°C (Brenner, 1974). The N2 (Bristol) strain was used as the WT. An unbiased genetic screen was performed using the NYL383 (*yadIs12[Pmec-4::GFP::unc-9 + Pttx-3::RFP] IV)* strain following the standard EMS mutagenesis protocol (Anderson, 1995). Mutant alleles used in this study include *unc-51(e1189)*, *unc-51(yad29)*, *unc-51(e369)*, *unc-76(e911)*, *unc-69(e587)*, *rab-10(dx2)*. A complete list of strains is described in Table S2.

### Plasmid construction and transgene generation

Plasmids were constructed using the ClonExpress recombination technology (C112-01; Vazyme) or Gateway cloning technology (311791020; Invitrogen). Gateway entry clones were generated using the backbone vector pCR8-JARID2 (114443; Addgene), and destination vectors were derived from pCZGY553 (110883; Addgene). Gene fragments were amplified from *C. elegans* N2 genomic DNA or cDNA.

For germline transformation, plasmids were microinjected at 10–50 ng/μl with coinjection markers (*Pttx-3::RFP*, *Prab-3::mCherry*, or *Podr-1::GFP*) at 10 ng/μl. Phosphomimetic UNC-76 variants (*Pmec-4::unc-76(S54D)* and *Pmec-4::unc-76(S54E)*) and dominant-negative RAB-10 (*Pmec-4::rab-10(T23N)*) were generated by site-directed mutagenesis of WT constructs using Vazyme Mut Express II Fast Mutagenesis Kit (C214-01). All constructs were sequence-verified before microinjection. A complete list of plasmids is provided in Table S1.

### CRISPR/Cas9-mediated gene editing

CRISPR/Cas9-mediated genome editing was performed following the self-excising cassette (SEC) method described by Dickinson et al. (2015). A donor plasmid was constructed using the pDD282 backbone (66823; Addgene), which contains the SEC. Homology arms (5′ and -3′) were amplified from N2 genomic DNA and cloned into pDD282 using the Gibson assembly (C112-01; Vazyme). Plasmids expressing the Cas9 protein and single-guide RNAs (sgRNAs) were derived from pDD162 (47549; Addgene) and generated using a site-directed mutagenesis kit (SMK-101; TOYOBO).

To generate the *unc-76* (caa97[*unc-76::mNeonGreen*]) knock-in strain, a microinjection mix containing donor plasmid (50 ng/μl; 852 bp 5′ homology arm + mNeonGreen + 669 bp 3′ homology arm), sgRNA-expressing plasmids (40 ng/μl; sgRNA#1: 5′-AAT​GTA​GTC​TGT​CAG​CAG​GG-3′; sgRNA#2: 5′-TAT​TGT​CAT​CGT​GGA​TTG​CG-3′), and Prab-3::mCherry coinjection marker (10 ng/μl; 19359; Addgene) was injected into young adult N2 hermaphrodites.

The *unc-9::47aa::mNeonGreen* and *unc-9::27aa::mNeonGreen* strains were generated using a similar strategy with sgRNA#1 (5′-CGT​ATG​GTT​GCA​ACT​CAC​GC-3′) and sgRNA#2 (5′-GAG​TTG​CAA​CCA​TAC​GAA​GT-3′), while *unc-9::4aa::mNeonGreen* was generated with sgRNA#1 (5′-ACG​AGC​TTG​CGA​AAA​CGT​TA-3′) and sgRNA#2 (5′-AAT​TGA​AAA​AGC​TAC​GAA​GT-3′). Injected worms and their progeny were selected with hygromycin B (250 µg/ml) to identify roller individuals carrying successful integrations. Hygromycin-resistant rollers were screened by PCR genotyping, and the SEC cassette was removed by heat shock at 34°C for 6 h. Nonroller homozygous knock-in worms were confirmed by PCR genotyping. sgRNA sequences and CRISPR-related primers are listed in Table S3.

### RNA interference

RNA interference (RNAi) was performed by feeding worms with *E. coli* strain HT115 expressing double-stranded RNA (dsRNA). RNAi clones from the Ahringer feeding library (Kamath, 2003) were cultured overnight in LB broth containing ampicillin (100 µg/ml) at 37°C. Bacterial cultures were concentrated and seeded onto NGM plates with ampicillin (100 µg/ml) and isopropyl 1-thio-β-D-galactopyranoside (5 mM), followed by overnight incubation at 37°C to induce dsRNA expression. For neuron-specific RNAi, the transgenic strain *sid-1*(*pk3321*)*; uIs69[Punc-119::sid-1]* was used, in which RNAi sensitivity is restored exclusively in neurons (Feinberg and Hunter, 2003; Calixto et al., 2010). *unc-22* RNAi was used as a positive control to assess RNAi efficacy. Worms were analyzed after being maintained on RNAi-expressing bacteria for at least two generations.

### HaloTag staining

For HaloTag::UNC-9 dynamic experiments, worms were incubated with 1 μM tetramethylrhodamine (TMR) HaloTag ligand (CA-TMR-1) in feeding culture at 20°C for 3 h in the dark. The feeding culture consisted of M9 buffer supplemented with centrifuged OP50 bacteria. Following incubation, worms were washed twice with M9 buffer and transferred to fresh NGM plates for 30 min at 20°C prior to imaging.

For time-lapse imaging of HaloTag::UNC-9 dynamics, L1 worms were immobilized and imaged on the spinning-disk microscope with 100× objective. Images were acquired every 1.0 s for 3.4 min. Anterograde and retrograde movement events were defined as the directional displacement of discrete HaloTag::UNC-9 puncta persisting for at least 10 frames. The proportion of anterograde and retrograde movement events among total scored puncta was calculated and quantified using ImageJ.

### Microscopy

Worms were immobilized on 6% agarose pads using either 1% 1-phenoxy-2-propanol (confocal imaging) or 2–8 mM levamisole (spinning-disk and wide-field imaging) in M9 buffer. Confocal images were acquired using a Zeiss LSM 880 microscope with a 40 × oil-immersion objective and ZEN Black acquisition software. Time-lapse and optogenetic ablation-related images were acquired using a Nikon CWU-1 spinning-disk microscope equipped with a Hamamatsu ORCA-Fusion camera and a 100× oil-immersion objective (200-ms exposure per channel), and a Live-SR module (GATACA Systems) that restricts excitation/photoactivation light to a user-defined region of interest (ROI), enabling spatially targeted illumination of selected PLM axonal segments. Image acquisition was controlled using MetaMorph software. Wide-field calcium imaging was performed using a Zeiss Axioscope 5 microscope with a 63 × oil-immersion objective (100-ms exposure, 20% LED illumination) and acquired by FCSnap software. Images were processed using ImageJ and Imaris Viewer.

### Calcium imaging and wavelet analysis

Imaging procedure followed established protocols (Chronis et al., 2007; Guo et al., 2009). GCaMP6s-expressing L1 or L4 hermaphrodites were immobilized on 5% agarose with 2 µM levamisole and mechanically stimulated by coverslip pressure. Changes were quantified as ΔF/F_0_ with 5-s baseline periods.

For wavelet-based signal analysis, the workflow was adapted from Péter and Héja (2024). ΔF/F_0_ traces were calculated from raw fluorescence intensity values. Wavelet decomposition was performed using a complex Morlet wavelet (bandwidth = 1; center frequency = 1). The secondary calcium signal energy proportion was quantified as the ratio of the summed energy of frequency components above 0.1 Hz within the secondary response window (5–30 s) to that of frequency components above 0.1 Hz across the full response duration (2–38 s).

### Dendra2 photoconversion

For Dendra2 photoconversion experiments, prephotoconversion images of PLM axons were acquired with both 488- and 561-nm channels (200-ms exposure). Photoconversion was performed by illuminating PLM axonal regions containing transient electrical synapses via the same Live-SR–based targeted illumination (see Microscopy) with 405-nm laser at 50% power for 20 s. Postphotoconversion imaging was immediately performed using identical settings. Worms were then transferred to NGM plates and maintained at 20°C. After 3 h, PLM axons were reimaged using the same parameters.

### miniSOG-mediated optogenetic ablation

For targeted miniSOG ablation, the strategy was adapted from previously described methods (Qi et al., 2012; Xu and Quinn, 2016). Synchronized L1-stage *yadIS12* worms expressing *Pmec-4::miniSOG::unc-9* were mounted on agar pads and exposed to blue light to induce ablation. PLM axonal regions containing transient electrical synapses were selectively illuminated via the Live-SR–based targeted illumination described above, using the 488-nm laser line at 2 Hz (250 ms ON/ 250 ms OFF) with 80% laser power for 4 min, while avoiding adjacent regions to minimize off-target photodamage. After illumination, animals were used for analyses or recovered from the agar pads and cultured under standard conditions.

### Image and quantitative analysis

For endogenous UNC-76 expression analysis in PLM cell bodies, the strain *unc-76(caa97[unc-76::mNeonGreen]*); *yadIs12[Pmec-7::mCherry*; *Pglr-1::mKate]* was used. The *unc-76::mNeonGreen* allele was generated by CRISPR/Cas9 knock-in, and the integrated *Pmec-7::mCherry* and *Pglr-1::mKate* transgenes facilitated PLM identification. Fluorescence images were acquired with 150-ms exposure and 80% LED illumination. The mNeonGreen fluorescence intensities were quantified using ImageJ. For each worm, three ROIs were selected within the PLM cell body, and mean fluorescence intensity was calculated. Background fluorescence from nonneuronal regions was subtracted to obtain corrected intensity values.

For Dendra2::UNC-9 fluorescence quantification, fold-change analysis was performed at three time points: before photoactivation, 0 h after photoactivation, and 3 h after photoactivation. ROIs were defined within the PLM cell body and along photoconverted neurites, with ROI position and size kept constant across time points. Dendra2 fluorescence intensities from both green and red channels were measured at each time point. For each biological replicate, fold change was calculated as the fluorescence intensity at postphotoactivation time points relative to the prephotoactivation value, which was set to 1 as the normalization baseline.

For UNC-51 expression analysis in the PLM cell bodies of *Punc-51::unc-51::mKate* worms, the same individual animals were imaged at both L1 and L4 stages. Three ROIs were selected within each PLM cell body, and the mean fluorescence intensity was measured. Background fluorescence from adjacent nonneuronal regions was subtracted to obtain corrected fluorescence intensity values.

For calcium imaging fluorescence quantification, ROIs were defined within PLM cell bodies, and fluorescence intensity was acquired across time. Background fluorescence from nonneuronal regions was subtracted to obtain corrected values.

For PLM axon extension quantification, axon length was defined as the distance from the distal tip of the anterior axon to the vulva. Analyzed images were acquired with CWU-1 spinning-disk microscope 100 × objective, and axon length was measured using ImageJ.

### Cold shock

The cold shock assay was performed based on the procedure previously described by Chatzigeorgiou et al. (2010). L1 larvae were incubated in M9 buffer supplemented with OP50 and exposed to repeated temperature cycles consisting of 1 min at 15°C followed by 3 min at 20°C for 6 h. After the cold shock treatment, worms were maintained at room temperature for an additional 27 h until reaching the L4 stage and were subsequently used for observation.

### Behavioral assays

Posterior mechanosensory responses were evaluated by applying a light tactile stimulus near the tail using an eyebrow hair (Chalfie, 2014). Animals exhibiting an immediate forward or backward locomotor response were scored as responsive. Three independent experiments were performed for each genotype.

### Statistical analysis

All data were analyzed using GraphPad Prism. Results are presented as the mean ± SEM. Prior to statistical analysis, all data were assessed for normality using the Shapiro–Wilk test and for variance homogeneity using the F test and Brown–Forsythe test. Statistical significance was determined using one-way or two-way analysis of variance (ANOVA), or Welch’s ANOVA when variances were unequal, followed by Tukey’s post hoc test for multiple comparisons, or by paired or two-tailed unpaired Student’s *t* test, one-sample *t* test, Wilcoxon’s matched-pairs signed-rank test, Mann–Whitney U test, or Kruskal–Wallis test, depending on the experimental design and data distribution. For all quantifications, P values are indicated in the figures, where *P < 0.05, **P < 0.01, ***P < 0.001, and n.s. indicates not significant (P > 0.05).

### Online supplemental material

Fig. S1 shows validation of transient electrical synapse dynamics with independent reporters. Fig. S2 shows controls for neurite morphology and chemical synapse assays. Fig. S3 shows genetic characterization of the UNC-51/UNC-76 pathway. Fig. S4 shows UNC-51 expression, *unc-76::mNeonGreen* knock-in validation, and Ser54 site identification. Fig. S5 shows quantification of UNC-9-UNC-76 cotransport events. Fig. S6 shows *rab-10* mutant phenotype and UNC-9-RAB-10 cotransport quantification. Table S1 lists the plasmids used in this study. Table S2 lists the *C. elegans* strains used in this study. Table S3 lists the CRISPR-related primers used in this study.

## Supplementary Material

Review History

Table S1 shows plasmids used in the study.

Table S2 shows *C. elegans* strains used in the study.

Table S3 shows CRISPR-related primers used in the study.

## Data Availability

The data supporting the findings of this study are available within the article and its online supplemental material. *C. elegans* strains and plasmids generated in this study are available from the corresponding author upon request. Raw imaging data underlying the quantifications presented in this study are available from the corresponding author upon reasonable request.
