# Supplementary material for: Developmental elimination of electrical synapses by UNC-51/UNC-76–mediated vesicular transport
Source: J Cell Biol. 2026 Nov 6;226(1):e202602115. doi: 10.1083/jcb.202602115 (PMC13741927; doi:10.1083/jcb.202602115)
Supplement: Supplementary file 1 [file jcb_202602115_review_history.pdf]

JCB

Developmental Elimination of Electrical Synapses by UNC-51/UNC-76-Mediated Vesicular Transport

Hao Huang, Yuzhi Yang, Yuanhao Xu, Yuli Jian, Shujie Qiu, Zihao Zhao, Dong Yan, and Lingfeng Meng

Corresponding Author(s): Lingfeng Meng, Zhejiang University-University of Edinburgh Institute

Review Timeline:

Submission Date:	2026-02-20
Editorial Decision:	2026-03-16
Revision Received:	2026-08-14
Editorial Decision:	2026-09-09
Revision Received:	2026-09-29

Monitoring Editor: Xiang Yu

Scientific Editor: Gabriele Stephan

Transaction Report:

(Note: With the exception of the correction of typographical or spelling errors that could be a source of ambiguity, letters and reports are not edited. The original formatting of letters and referee reports may not be reflected in this compilation.)

DOI: https://doi.org/10.1083/jcb.202602115

March 16, 2026

Re: JCB manuscript #202602115

Lingfeng Meng
Zhejiang University-University of Edinburgh Institute

Dear Dr. Meng,

Thank you for submitting your manuscript entitled "Developmental Elimination of Electrical Synapses by UNC-51/UNC-76-Mediated Vesicular Transport". Your manuscript has been assessed by expert reviewers, whose comments are appended below. Although the reviewers express potential interest in this work, significant concerns unfortunately preclude publication of the current version of the manuscript in JCB.

You will see that all reviewers think your paper provides interesting findings towards the developmental elimination of electrical synapses, while offering constructive feedback. We agree with this assessment, and we think your paper could improve if you were able to fully address the reviewers' concerns. We believe that it is especially critical to demonstrate genetic relationship between UNC-51 and UNC-76 or the UNC-51-UNC-76-RAB-10 axis, as voiced by reviewers #1 and #3, and present more in-depth mechanistic insights including those underlying the causal relationship between electrical synapses, calcium oscillations, and chemical synapse formation. Reviewer #3's requirement for data to identify transient vs. stable gap junction partners in PLM neurons as well as stabilized gap junctions in mutant backgrounds is also critical to the interpretation of the phenotypes presented, in a developmental context. Reviewer #2's suggestions as well as all requested control experiments and textual alterations should further be included in a revised manuscript.

Please let us know if you are able to address the major issues outlined above and wish to submit a revised manuscript to JCB. Note that a substantial amount of additional experimental data likely would be needed to satisfactorily address the concerns of the reviewers. It may be necessary to extend your manuscript to a full Research Article. The typical timeframe for revisions is three to four months. If you anticipate any difficulties in meeting this aforementioned revision time limit, please contact us and we can work with you to find an appropriate time frame for resubmission. Please note that papers are generally considered through only one revision cycle, so any revised manuscript will likely be either accepted or rejected.

If you choose to revise and resubmit your manuscript, please also attend to the following editorial points. Please direct any editorial questions to the journal office.

GENERAL GUIDELINES:
Text limits: Character count for a Report is < 20,000; a full Research Article is < 40,000, not including spaces. Count includes title page, abstract, introduction, the joint Results & Discussion, and acknowledgments. Count does not include materials and methods, figure legends, references, tables, or supplemental legends.

Figures: A Report may include up to 5 main text figures; a full Research Article may have up to 10 main text figures. To avoid delays in production, figures must be prepared according to the policies outlined in our Instructions to Authors, under Data Presentation, https://jcb.rupress.org/site/misc/ifora.xhtml. All figures in accepted manuscripts will be screened prior to publication.

IMPORTANT: It is JCB policy that if requested, original data images must be made available. Failure to provide original images upon request will result in unavoidable delays in publication. Please ensure that you have access to all original microscopy and blot data images before submitting your revision.

Supplemental information: There are strict limits on the allowable amount of supplemental data. Reports may have up to 3 supplemental figures; a full Research Article may have up to 5 supplemental figures. Up to 10 supplemental videos or flash animations are allowed. A summary of all supplemental material should appear at the end of the Materials and methods section.

Please note that JCB now requires authors to submit Source Data used to generate figures containing gels and Western blots with all revised manuscripts. This Source Data consists of fully uncropped and unprocessed images for each gel/blot displayed in the main and supplemental figures. For assays performed using capillary electrophoresis and/or immunoassay-based detection, authors should instead provide the electropherogram graph(s) for each experiment, plotting fluorescence/chemiluminescence intensity vs. molecular weight/size. Please be sure to provide one Source Data file for each figure gels, blots, and/or capillary electrophoresis assays along with your revised manuscript files. File names for Source Data figures should be alphanumeric without any spaces or special characters (i.e., SourceDataF#, where F# refers to the associated main figure number or SourceDataFS# for those associated with Supplementary figures). For traditional gels and blots, the lanes of the gels/blots should be labeled as they are in the associated figure, the place where cropping was applied should be marked (with a box), and molecular weight/size standards should be labeled wherever possible. For capillary electrophoresis

assays, each trace in the graph should be color-coded and labeled to indicate which protein, gene, or sample is being measured (please try to avoid red/green combinations to accommodate our color-blind readers).

Source Data files will be made available to reviewers during evaluation of revised manuscripts and, if your paper is eventually published in JCB, the files will be directly linked to specific figures in the published article.

Source Data Figures should be provided as individual PDF files (one file per figure). Authors should endeavor to retain a minimum resolution of 300 dpi or pixels per inch. Please review our instructions for export from Photoshop, Illustrator, and PowerPoint here: https://rupress.org/jcb/pages/submission-guidelines#revised

If you choose to resubmit, please include a cover letter addressing the reviewers' comments point by point. Please also highlight all changes in the text of the manuscript.

Regardless of how you choose to proceed, we hope that the comments below will prove constructive as your work progresses. We would be happy to discuss them further once you've had a chance to consider the points raised. You can contact the journal office with any questions at cellbio@rockefeller.edu.

Thank you for thinking of JCB as an appropriate place to publish your work.

Sincerely,

Xiang Yu
Monitoring Editor
Journal of Cell Biology

Gabriele Stephan
Scientific Editor
Journal of Cell Biology

Reviewer #1:

This manuscript by Huang et al. investigates how transient electrical synapses are eliminated during neural circuit maturation using C. elegans PLM mechanosensory neurons. The authors report that electrical synapses form transiently during early development and promote high-frequency calcium oscillations, which are proposed to influence chemical synapse formation. Furthermore, the study identifies a pathway involving the conserved kinase UNC-51/ULK, the kinesin adaptor UNC-76/FEZ, and RAB-10 that regulates retrograde trafficking of innexins and elimination of electrical synapses. Generally, the topic is interesting and addresses an important question regarding the developmental relationship between electrical and chemical synapses. The identification of a molecular mechanism for electrical synapse elimination is potentially significant. However, several key conclusions are currently insufficiently supported by the data, and additional experiments are needed to establish the causal relationships proposed in the manuscript.

Major Concerns
1. Lack of causal relationship between electrical synapses, calcium oscillations, and chemical synapse formation. A central claim of the manuscript is that transient electrical synapses generate high-frequency calcium oscillations and promote chemical synapse formation. However, the current data largely show correlations rather than causality.

For example, the authors report that L1 neurons display both transient electrical synapses and stronger high-frequency calcium oscillations compared with L4 neurons (Fig. 2A, E). However, this comparison alone does not demonstrate that electrical synapses directly generate the oscillations. Similar language appears in the Discussion ("Transient electrical synapses in PLM neurons generate high-frequency calcium oscillations"), which implies a causal relationship that is not directly demonstrated.

To support this model, additional experiments would be helpful, for example, examining calcium oscillations in unc-51 mutants that disrupt electrical synapses, testing whether manipulating calcium dynamics (like optogenetics) restore electrical synapse elimination or chemical synapse formation.

2. The authors propose that UNC-51 phosphorylates UNC-76 to promote retrograde trafficking of innexins. However, the genetic relationship between these components requires stronger support. Specifically, does expression of UNC-76(S54E) rescue the calcium oscillation phenotype observed in unc-51 mutants? Analysis of unc-51; unc-76 double mutants would help determine whether the two genes function in the same genetic pathway.

3. Further, in Fig. 3D, the unc-51 phenotype appears stronger than the unc-76 phenotype, despite UNC-76 being proposed as a

downstream effector. This suggests that UNC-51 may have additional targets in this pathway. The observation that UNC-76(S54E) only partially rescues unc-51 defects would be consistent with this possibility. Further analysis would strengthen the proposed signaling pathway.

4. Lack of evidence that UNC-9 puncta represent functional electrical synapses. The study relies heavily on UNC-9 puncta as a marker of electrical synapses. However, it is not clear whether these puncta represent functional gap junctions or simply the localization or turnover of innexin proteins. Additional evidence would strengthen this interpretation. For example, colocalization of UNC-9 with UNC-7.

5. The manuscript proposes that UNC-51-UNC-76 signaling promotes RAB-10-dependent retrograde trafficking of UNC-9. However, the trafficking analysis focuses mainly on co-transport events. It would be important to clarify: what fraction of total UNC-9 transport events involve co-transport with the proposed machinery? whether overall anterograde and retrograde trafficking of UNC-9 is altered in the mutants? This information would help evaluate the overall impact of the proposed pathway on UNC-9 trafficking.

6. The proposed elimination mechanism appears to operate specifically during early development (L1 stage). However, the basis for this developmental regulation remains unclear. It would be informative to examine whether UNC-51 expression or activity changes during development, or whether other regulatory mechanisms control the timing of electrical synapse elimination.

Minor
1. The developmental reduction of electrical synapses in ALM neurons appears less clear than in PLM neurons. Additional quantification or representative images would help clarify this point.

2. In Fig. 1F-G, representative images of the cell body before photoconversion and at 0 and 3 hours after photoconversion are not shown.

3. In unc-76 mutants, UNC-9 puncta appear reduced already at the L1 stage, and this issue should be clarified.

4. The manuscript states that autophagy mutants do not reproduce the electrical synapse phenotype ("data not shown"). Because this conclusion is important for excluding an autophagy mechanism, the relevant data should be included in the supplementary information.

5. The physiological meaning of the observed high-frequency calcium oscillations remains unclear and could be discussed more explicitly.

6. If electrical synapses fail to be eliminated and persist into later developmental stages, it would be interesting to examine whether this affects mechanosensory behavior or circuit function.

Reviewer #2:

This study demonstrates that transient electrical synapses promote early calcium spikes during neuronal development that are important for proper synaptogenesis. The authors show that these immature electrical synapses are progressively eliminated through retrograde transport of the innexin UNC-9. Through a forward mutagenesis screen and genetic analyses, they propose that UNC-9 removal is mediated by a retrograde transport pathway involving UNC-51, UNC-76, and the vesicle trafficking factor RAB-10. Based on these findings, the authors propose a model in which early electrical synapses generate calcium influx that facilitates chemical synapse formation, after which the gap junction components are removed through a retrograde recycling mechanism during circuit maturation. The conceptual framework linking transient electrical synapses, calcium signaling, and synapse maturation will likely be of interest to readers of the Journal of Cell Biology. The authors should consider addressing several suggestions before the manuscript can be considered for publication.
1. I do not agree with the interpretation of Figure 5. First "Quantitative analysis revealed that transport patterns were consistent across developmental stages. In L1, anterograde co-transport occurred in 65% of cases, while L4 animals showed in 61% of cases, with no significant difference between stages (Fig. 5B). These findings demonstrate that UNC-76 directly participates in the bidirectional axonal transport of innexins." I think that this only suggests UNC-76 and UNC-9 are co-transported in both directions. (This is actually further substantiated by the authors showing that the mutation of unc-76 did not impact the transportation direction at all). The authors show that overexpression of the phosphomimetic form of UNC-76 slightly increased UNC-9 retrogradation and suggested that phosphorylated UNC-76 drives retrograde. However, I do not think the evidence is solid enough to support the author's hypothesis. Do the authors consider the alternative hypothesis that UNC-51-UNC-76-RAB-10 machinery actually facilitates the endocytosis of the UNC-9 from the membrane, and the increase in retrograde transportation (when overexpressed UNC-76S54E) is the consequence of the influx of increased endocytosed UNC-9?
2. Using the Dendra2 photobleaching method, the authors found that red signals increase in the cell soma after 3 hours, which is a critical first piece of evidence that the UNC-9 retrograde transport mechanism exists. I suggest that the authors elaborate on

this hypothesis earlier. And the authors should also show the representative images of soma in the Figure 1 too.
3. I also suggest that the authors perform the tracing of photoconverted UNC-9 to establish the hypothesis of whether the transient gap junctions show a higher probability of retrograde transport back to the soma.
4. "Given the rapid elimination in electrical synapse at early stages, we performed time-lapse imaging of HaloTag::UNC-9 in L1 PLM neurons to examine its dynamic behavior. This analysis revealed that transient electrical synapses undergo continuous assembly and disassembly (Fig S1). The authors did not provide sufficient information on the methods in text, and there is no image in Fig. S1B; thus, I have a hard time understanding the interpretation.
5. The authors suggests that calcium influx through transient electrical synapses is crucial for chemical synapse formation. Is it sufficient to promote chemical synapses? Did the authors check in the unc-51 or unc-76 mutant whether there is ectopic/increased synaptic formation?

Minor revision:
1."Although gap junctions are known to transmit calcium and second messengers, whether these signals causally instruct chemical synapse development-and how these transiently essential connections are themselves subsequently eliminated-remain entirely unknown." The cited citations have already implicated the hypothesis. I suggest to use "remain mechanistically unclear"
2. Figure 3D required a wild-type control for proper comparison.
3. The authors should also indicate N in every graph for the reader to easily access.
4. Page 9 "Remarkably, expression of UNC-76(S54E) in unc- 76(e911) mutant worms partially rescued the excessive electrical synapse phenotypes." Should it be unc-51?

Reviewer #3:

Huang et al. demonstrate that fluorescence labeled innexin proteins form transient clusters on the PLM process during early development, which subsequently disappear as the animals mature. Disruption of these transient innexin clusters leads to a reduction in later-developing chemical synapses, accompanied by diminished high-frequency calcium oscillations. The authors further identify that UNC-51/ULK is required for this process. Previous studies have suggested that UNC-51 may regulate UNC-76/FEZ, which in turn links to UNC-69. Based on this, phenotypic analysis hinted that UNC-76 and UNC-69 may function in conjunction with UNC-51 to regulate the elimination of transient gap junctions.
Major Points:
1. Identification of transient vs. stable GJ partners: Neural circuit re-wiring during development is a well-established phenomenon in the C. elegans nervous system. The transiently formed innexin clusters described here likely represent neuronal connections with transient developmental partners. Moreover, gap junction (GJ) formation could be influenced by both sides of the partnering cells. Therefore, it is critical to determine whether the synaptic partners of PLM in early (L1) stages differ from those in later stages. If so, disruption of these transient innexin clusters may damage PLM-related connectivity indirectly-by affecting upstream developmental events such as cell migration or axon guidance-rather than directly impacting the formation of chemical synapses or calcium signals. To strengthen the significance of transient GJ elimination, the authors should identify the electrical synapse partners of PLM, distinguishing between stable connections (e.g., those near the cell body) and transient ones. Notably, GJ connections such as PLM-BDU have been previously characterized; similar experimental approaches could be employed to systematically identify those GJ partners of PLM.

2. Characterization of stabilized GJ in unc-51 and other mutants: The transient GJs appear abnormally stabilized in unc-51 mutants. Without identifying the specific PLM GJ partners in both wild-type and unc-51 mutant backgrounds, it remains unclear whether the abnormally stabilized GJ clusters in unc-51 mutants represent the same transient partnerships seen in wild-type animals or entirely different categories of junctions. This ambiguity undermines the core conclusion that UNC-51 is specifically involved in eliminating transient GJs. The same concern applies to analyses of unc-76, unc-69, and rab-10; the authors should identify the GJ partners of PLM under each genetic condition before drawing meaningful conclusions about their roles in GJ elimination.

3. Clarification of UNC-51/UNC-76 genetic interaction: The proposed regulatory relationship between UNC-51 and UNC-76 is confusing and, in places, appears to conflict with the presented data. Although unc-76 mutants exhibit phenotypes similar to unc-51, the observation that UNC-76 expression is five-fold higher in unc-51 mutants than in wild-type directly contradicts the conclusion that UNC-51 acts upstream to positively regulate UNC-76 function in GJ elimination. Did the authors examine GJ phenotypes using a gain-of-function allele of unc-76? Alternatively-and perhaps more plausibly-UNC-76 and UNC-51 may act in parallel pathways or distinct biological processes to regulate GJ formation. This alternative interpretation should be considered and investigated.

4. The phenotypic similarity alone is insufficient to support the proposed linear regulatory cascade from UNC-51 to UNC-76 to

UNC-69 to RAB-10. The current data primarily rely on the so-called stabilized "transient gap junction" as a readout. While loss-of-function of the above genes all display similar phenotypes, this is not sufficient evidence to establish a sequential regulation cascade. More direct and insightful mechanistic links should be provided.

Minor Points:

1. Discrepancy in rescue experiment description: The description of the unc-76 rescue experiments is inconsistent and requires clarification. In the text, the authors state: "expression of UNC-76(S54E) in unc-76(e911) mutant worms partially rescued the excessive electrical synapse phenotypes, whereas wild-type UNC-76 expression showed no rescue effect (Fig. 4C,D)." However, Figures 4C and 4D indicate that unc-76 expression was actually performed in an unc-51 mutant background. This discrepancy must be resolved.

2. Missing citations: The manuscript appears to omit several relevant citations, particularly previous findings on how gap junctions influence chemical synapse formation during early nervous system development, as well as established knowledge (and gaps in knowledge) regarding the GJ partners of PLM in C. elegans. These references should be incorporated to properly contextualize the findings.

Reviewer #1:

This manuscript by Huang et al. investigates how transient electrical synapses are eliminated during neural circuit maturation using C. elegans PLM mechanosensory neurons. The authors report that electrical synapses form transiently during early development and promote high-frequency calcium oscillations, which are proposed to influence chemical synapse formation. Furthermore, the study identifies a pathway involving the conserved kinase UNC-51/ULK, the kinesin adaptor UNC-76/FEZ, and RAB-10 that regulates retrograde trafficking of innexins and elimination of electrical synapses. Generally, the topic is interesting and addresses an important question regarding the developmental relationship between electrical and chemical synapses. The identification of a molecular mechanism for electrical synapse elimination is potentially significant. However, several key conclusions are currently insufficiently supported by the data, and additional experiments are needed to establish the causal relationships proposed in the manuscript.

Major Concerns

1. Lack of causal relationship between electrical synapses, calcium oscillations, and chemical synapse formation. A central claim of the manuscript is that transient electrical synapses generate high-frequency calcium oscillations and promote chemical synapse formation. However, the current data largely show correlations rather than causality.

For example, the authors report that L1 neurons display both transient electrical synapses and stronger high-frequency calcium oscillations compared with L4 neurons (Fig. 2A, E). However, this comparison alone does not demonstrate that electrical synapses directly generate the oscillations. Similar language appears in the Discussion ("Transient electrical synapses in PLM neurons generate high-frequency calcium oscillations"), which implies a causal relationship that is not directly demonstrated.

To support this model, additional experiments would be helpful, for example, examining calcium oscillations in unc-51 mutants that disrupt electrical synapses, testing whether manipulating calcium dynamics (like optogenetics) restore electrical synapse elimination or chemical synapse formation.

We agree that fully establishing causality is difficult with imaging alone. Our original submission already showed that optogenetic ablation of transient electrical synapses reduces high-frequency calcium oscillations and increases chemical synapse defects, and that unc-51 mutants exhibit elevated oscillations at L4. Addressing the reviewer's two suggestions, we have now added two new experiments: ablating the persistent gap junctions specifically in unc-51 mutants, which significantly reduced their elevated oscillations; and restoring calcium activity via TRPA-1 in electrical synapse-ablated animals, which substantially rescued chemical synapse formation. Together, these new results—combined with our original ablation experiments—support a causal chain linking electrical synapse activity, calcium oscillations, and chemical synapse formation; full details are provided in the revised Results (Fig. 2, Fig. 3) and Discussion.

2. The authors propose that UNC-51 phosphorylates UNC-76 to promote retrograde trafficking of

innexins. However, the genetic relationship between these components requires stronger support. Specifically, does expression of UNC-76(S54E) rescue the calcium oscillation phenotype observed in unc-51 mutants? Analysis of unc-51; unc-76 double mutants would help determine whether the two genes function in the same genetic pathway.

We performed additional experiments to directly address this question. Calcium imaging in *unc-51;unc-76* double mutants at L4 showed energy proportion levels comparable to either single mutant alone (Fig. 3G,I), consistent with UNC-51 and UNC-76 acting within the same genetic pathway. Furthermore, expression of phosphomimetic UNC-76(S54E) in unc-51 mutants significantly suppressed the elevated energy proportion toward wild-type levels (Fig. 4I), directly demonstrating that UNC-51-mediated phosphorylation of UNC-76 at Ser54 is required not only for electrical synapse elimination but also for the consequent termination of high-frequency calcium oscillations.

3. Further, in Fig. 3D, the unc-51 phenotype appears stronger than the unc-76 phenotype, despite UNC-76 being proposed as a downstream effector. This suggests that UNC-51 may have additional targets in this pathway. The observation that UNC-76(S54E) only partially rescues unc-51 defects would be consistent with this possibility. Further analysis would strengthen the proposed signaling pathway.

We agree, and had noted this ourselves: the stronger phenotype of unc-51 mutants relative to unc-76 mutants, together with the partial rescue by UNC-76(S54E), suggests that UNC-51 likely has additional substrates in this pathway. This would be consistent with the broad substrate repertoire of UNC-51/ULK1 across species, which regulates diverse processes including autophagy (Russell et al., 2013; Di Bartolomeo et al., 2010; Papinski et al., 2014), innate immune signaling (Konno et al., 2013), and axonal transport via UNC-76/FEZ1 phosphorylation—the same relationship characterized here (Toda et al., 2008). Although our candidate-based genetic and RNAi suppressor screens did not identify additional components beyond UNC-76, these approaches have inherent coverage limitations, and additional UNC-51 substrates contributing to electrical synapse elimination likely remain to be identified.

Although the UNC-51-mediated phosphorylation of UNC-76 has been established in *Drosophila*, we sought to directly reproduce this biochemical relationship in *C. elegans*. Because no phospho-specific antibody against UNC-76 is available for C. elegans, we employed an antibody-independent, Phos-tag-based phosphorylation assay (Phos-assay Acrylamide, Vazyme, PA101-01), based on the principle described by Kinoshita et al. (2006). However, we were unable to detect a phosphorylation signal for UNC-76 under our experimental conditions, likely due to the low abundance of phosphorylated UNC-76 in animals. We therefore sought other evidence to support the UNC-51–UNC-76 interaction. Time-lapse imaging revealed co-localization of UNC-51 and UNC-76 in PLM neurons, consistent with these proteins functioning in the same complex. Furthermore, UNC-51 co-localizes with UNC-9 puncta along PLM axons, placing the kinase in direct proximity to its proposed site of action. Together, these observations provide indirect but

consistent support for UNC-51-mediated regulation of UNC-76 at electrical synapses.

4. Lack of evidence that UNC-9 puncta represent functional electrical synapses. The study relies heavily on UNC-9 puncta as a marker of electrical synapses. However, it is not clear whether these puncta represent functional gap junctions or simply the localization or turnover of innexin proteins. Additional evidence would strengthen this interpretation. For example, colocalization of UNC-9 with UNC-7.

We provide several lines of evidence supporting that UNC-9 puncta represent functional electrical synapses rather than non-functional protein aggregates.

First, GFP::UNC-9 puncta in PLM neurons were previously validated as gap junction plaques by correlative electron microscopy (Meng et al., 2016). Second, our original submission already showed that endogenous CRISPR-tagged UNC-9::mNeonGreen displays developmental dynamics identical to the transgenic GFP::UNC-9 reporter (Fig. 1C, S1A), confirming these puncta reflect endogenous localization rather than overexpression artifacts. To further strengthen this interpretation, we have now performed two additional experiments. We found co-localization of UNC-9 with UNC-7 at both transient (L1) and stable (L1 &L4) puncta along PLM neurites (Fig. 1E and Fig. S1C), consistent with heteromeric channel formation—a hallmark of functional gap junctions. Using a Cre-loxP-based cell-specific excision approach, we further found that transient UNC-9 puncta depend on the presence of a partnering cell, confirming they represent genuine intercellular junctions rather than non-specific aggregates (Fig. S1D,E). Subsequent co-localization analysis showed partial overlap between these puncta and the hypodermal (epithelial) innexin INX-9, suggesting that this partnering cell is likely epithelial in nature (Fig. S1G).

Together, these results support that UNC-9 puncta represent genuine transient gap junctions.

5. The manuscript proposes that UNC-51-UNC-76 signaling promotes RAB-10-dependent retrograde trafficking of UNC-9. However, the trafficking analysis focuses mainly on co-transport events. It would be important to clarify: what fraction of total UNC-9 transport events involve co-transport with the proposed machinery? whether overall anterograde and retrograde trafficking of UNC-9 is altered in the mutants? This information would help evaluate the overall impact of the proposed pathway on UNC-9 trafficking.

Co-transport imaging captures the cell-biological signature of this mechanism at a given imaging timepoint, rather than quantifying what fraction of the overall elimination process the pathway accounts for. Approximately 7% of total UNC-9 movements co-transported with UNC-76, and approximately 2% with RAB-10. It is important to note that gap junction maintenance and elimination occur simultaneously in PLM neurons, and that PLM neurons form both transient gap junctions along the posterior neurite and stable gap junctions at the anterior process with BDU interneurons. Our transport analysis therefore captures a mixed population of UNC-9 trafficking events reflecting both ongoing maintenance of stable junctions and active elimination of transient ones, which necessarily limits the fraction of total transport events that can be attributed

specifically to the elimination machinery.

Regarding whether overall UNC-9 transport directionality is altered in the mutants: in *unc-76* loss-of-function mutants, the retrograde-to-anterograde ratio of UNC-9 movements was indistinguishable from wild-type (Fig. 5C). We note that UNC-76 constitutively co-transports with UNC-9 in both directions (Fig. 5A,B), consistent with a continuous role in gap junction delivery and maintenance; the unchanged ratio in *unc-76* mutants therefore indicates that this constitutive, unphosphorylated activity is direction-neutral, rather than indicating that UNC-76 is dispensable for UNC-9 trafficking altogether. By contrast, expressing phosphomimetic UNC-76(S54E) significantly shifted this ratio toward retrograde movement in both wild-type and *unc-76* mutant backgrounds. Together, these results indicate that UNC-51-mediated phosphorylation converts UNC-76 from a direction-neutral participant in constitutive innexin trafficking into an active driver of net retrograde removal.

6. The proposed elimination mechanism appears to operate specifically during early development (L1 stage). However, the basis for this developmental regulation remains unclear. It would be informative to examine whether UNC-51 expression or activity changes during development, or whether other regulatory mechanisms control the timing of electrical synapse elimination.

We have addressed this directly. Using a *Punc-51::unc-51::mKate* reporter, we found no significant difference in UNC-51 expression between L1 and L4 (Fig. S4A,B), ruling out changes in overall expression as the explanation. However, PLM-specific imaging revealed a developmental shift in UNC-51 subcellular localization: at L1 it co-localized with both transient and stable gap junctions, but by L4 it was largely restricted to stable junctions (Fig. 4A,B). Combined with our finding that phosphorylated UNC-76 drives retrograde innexin removal, we propose that UNC-51's spatial enrichment at transient junctions specifically during L1 confers the elimination efficiency needed for developmental specificity, without requiring any change in its overall expression level. This provides a spatial, rather than expression-based, explanation for the timing of elimination; full details are in the revised Results (Fig. 4A,B,S4A,B) and Discussion.

Minor

1. The developmental reduction of electrical synapses in ALM neurons appears less clear than in PLM neurons. Additional quantification or representative images would help clarify this point.

We have replaced the representative images.

2. In Fig. 1F-G, representative images of the cell body before photoconversion and at 0 and 3 hours after photoconversion are not shown.

We have outlined the boundary of the cell soma in the corresponding figure panels for clarity.

3. In unc-76 mutants, UNC-9 puncta appear reduced already at the L1 stage, and this issue should be clarified.

UNC-76 participates in bidirectional innexin transport in its unphosphorylated state (Fig. 5), and its

loss may impair anterograde delivery of innexins, thereby reducing gap junction number already at L1 through a defect in assembly rather than premature elimination. This is consistent with our model in which UNC-51-mediated phosphorylation of UNC-76 shifts the balance toward retrograde transport—phosphorylation alters directionality bias rather than UNC-76's general role in innexin trafficking.

4. The manuscript states that autophagy mutants do not reproduce the electrical synapse phenotype ("data not shown"). Because this conclusion is important for excluding an autophagy mechanism, the relevant data should be included in the supplementary information.

We have included the corresponding data as a supplementary figure (Fig. S3B).

5. The physiological meaning of the observed high-frequency calcium oscillations remains unclear and could be discussed more explicitly.

We interpret these high-frequency calcium oscillations as a form of stage-specific spontaneous neural activity that provides an activity-dependent instructive signal for circuit assembly, rather than simply reflecting elevated neuronal excitability. This interpretation is directly supported by our TRPA-1 rescue experiments (Fig. 2J,K), which show that restoring high-frequency calcium activity after electrical synapse ablation is sufficient to substantially rescue the resulting chemical synapse defects. This places the PLM oscillations within a broader class of developmentally restricted spontaneous activity patterns—such as retinal waves, the elimination of polyneuronal innervation at the neuromuscular junction, and the closing of cortical critical periods—that instruct axon growth, synapse formation, and circuit refinement before being appropriately terminated. We have expanded the Discussion to make this point more explicit.

6. If electrical synapses fail to be eliminated and persist into later developmental stages, it would be interesting to examine whether this affects mechanosensory behavior or circuit function.

We examined gentle touch responses in pathway mutants. *unc-51* mutants could not be reliably scored: although PLM touch responsiveness itself was intact, their severe locomotor defects made it impossible to distinguish forward from backward movement. We therefore focused on *unc-76* mutants, whose locomotor defects are much milder. Touch responsiveness in *unc-76* mutants was normal, and most animals still escaped forward; however, a significant fraction showed inappropriate backward movement after posterior touch, a phenotype essentially absent in wild-type controls (Fig. S3J).

To obtain evidence unconfounded by locomotor defects, we used miniSOG to acutely and specifically eliminate transient electrical synapses at L1 and assayed touch responses at L4. Ablated animals showed a significantly higher proportion of touch response abnormalities than controls, directly demonstrating that transient electrical synapses are required for normal mechanosensory circuit development (Fig. 2I).

Regarding mechanism, PLM drives the forward escape response chiefly through its chemical

synapse onto PVC, so the miscoordination in *unc-76* mutants is consistent with either direct signal noise from persistent calcium oscillations or indirect disruption of PLM-PVC chemical synapse maturation—consistent with our finding that PLM chemical synapse labeling in both mutants showed an elevated proportion of synaptic abnormalities. We cannot yet fully disentangle these direct and indirect contributions; we are performing further locomotion pattern analyses in *unc-76* mutants to address this and will include the results in the revised manuscript.

Reviewer #2:

This study demonstrates that transient electrical synapses promote early calcium spikes during neuronal development that are important for proper synaptogenesis. The authors show that these immature electrical synapses are progressively eliminated through retrograde transport of the innexin UNC-9. Through a forward mutagenesis screen and genetic analyses, they propose that UNC-9 removal is mediated by a retrograde transport pathway involving UNC-51, UNC-76, and the vesicle trafficking factor RAB-10. Based on these findings, the authors propose a model in which early electrical synapses generate calcium influx that facilitates chemical synapse formation, after which the gap junction components are removed through a retrograde recycling mechanism during circuit maturation. The conceptual framework linking transient electrical synapses, calcium signaling, and synapse maturation will likely be of interest to readers of the Journal of Cell Biology. The authors should consider addressing several suggestions before the manuscript can be considered for publication.

1. I do not agree with the interpretation of Figure 5. First "Quantitative analysis revealed that transport patterns were consistent across developmental stages. In L1, anterograde co-transport occurred in 65% of cases, while L4 animals showed in 61% of cases, with no significant difference between stages (Fig. 5B). These findings demonstrate that UNC-76 directly participates in the bidirectional axonal transport of innexins." I think that this only suggests UNC-76 and UNC-9 are co-transported in both directions. (This is actually further substantiated by the authors showing that the mutation of unc-76 did not impact the transportation direction at all). The authors show that overexpression of the phosphomimetic form of UNC-76 slightly increased UNC-9 retrogradation and suggested that phosphorylated UNC-76 drives retrograde. However, I do not think the evidence is solid enough to support the author's hypothesis. Do the authors consider the alternative hypothesis that UNC-51-UNC-76-RAB-10 machinery actually facilitates the endocytosis of the UNC-9 from the membrane, and the increase in retrograde transportation (when overexpressed UNC-76S54E) is the consequence of the influx of increased endocytosed UNC-9?

We have revised our model accordingly, proposing that RAB-10 facilitates UNC-9 internalization from the synaptic membrane, after which phosphorylated UNC-76 mediates retrograde transport of RAB-10-positive endosomes away from the synapse. We have softened the mechanistic claims in both the Results and Discussion sections accordingly. Formally distinguishing endocytic internalization from direct retrograde transport remains technically challenging in the extremely fine neuronal processes of *C. elegans*, and we acknowledge this as an important question for future investigation.

2. Using the Dendra2 photobleaching method, the authors found that red signals increase in the cell soma after 3 hours, which is a critical first piece of evidence that the UNC-9 retrograde transport mechanism exists. I suggest that the authors elaborate on this hypothesis earlier. And the authors should also show the representative images of soma in the Figure 1 too.

We agree. We have moved the rationale for these experiments earlier in the Results, before the

HaloTag and Dendra2 experiments, and have labeled the soma position in the Figure 1 panels.

3. I also suggest that the authors perform the tracing of photoconverted UNC-9 to establish the hypothesis of whether the transient gap junctions show a higher probability of retrograde transport back to the soma.

Dynamic transport analysis places stringent demands on fluorescent protein brightness and photostability—while GFP and mNeonGreen perform reliably in our imaging conditions, we were unable to identify a red fluorescent protein with comparable performance for this purpose, which is also why we adopted HaloTag for our transport studies. Unfortunately, Dendra2 does not meet the technical requirements for dynamic transport analysis under our experimental conditions.

As an alternative, we quantified the distribution of red Dendra2 signal across the entire PLM neuron at 3 hours post-photoconversion, encompassing the axon, stable gap junction sites, and the cell soma. Red fluorescence was predominantly enriched in the soma, with relatively weak signal remaining at stable gap junction sites and along the axon. Although this approach cannot directly resolve individual UNC-9 transport trajectories, the distribution pattern is consistent with retrograde movement of UNC-9 from axonal regions back to the cell body, providing indirect support for the retrieval of transient gap junction components.

4. "Given the rapid elimination in electrical synapse at early stages, we performed time-lapse imaging of HaloTag::UNC-9 in L1 PLM neurons to examine its dynamic behavior. This analysis revealed that transient electrical synapses undergo continuous assembly and disassembly (Fig S1). The authors did not provide sufficient information on the methods in text, and there is no image in Fig. S1B; thus, I have a hard time understanding the interpretation.

We apologize for the lack of clarity here. In response to a related comment from another reviewer, we have restructured this paragraph so that the hypothesis is stated upfront, before the data are presented, and have reorganized the corresponding figure panels into Fig. S1H and S1I, with the imaging parameters—interval, duration, and the criteria used to score movement direction—now specified in the Methods.

5. The authors suggests that calcium influx through transient electrical synapses is crucial for chemical synapse formation. Is it sufficient to promote chemical synapses? Did the authors check in the unc-51 or unc-76 mutant whether there is ectopic/increased synaptic formation?

To address whether high-frequency calcium oscillations are sufficient to promote chemical synapse formation, we restored calcium activity independently of gap junction coupling using TRPA-1-mediated cold stimulation after miniSOG ablation. This significantly reduced the proportion of PLM neurons with chemical synapse defects compared to ablated controls (Fig. 2J,K), though the rescue was partial, suggesting additional calcium-independent signals likely also contribute.

To test whether persistent electrical synapses cause ectopic or increased chemical synapse formation, we examined *unc-51* and *unc-76* mutants. We did not observe ectopic or increased

synapse formation; instead, both mutants showed a significantly elevated proportion of PLM neurons with absent or abnormal chemical synapses (Fig. S3H,I). We note this result should be interpreted with caution, as UNC-51 and UNC-76 also regulate general vesicular transport.

Together, these results indicate a biphasic requirement for electrical synapse activity—both premature loss and persistence of electrical synapses disrupt chemical synapse maturation, consistent with precise temporal windows of activity required in other developmental contexts. We have expanded the Discussion to address this point explicitly

Minor revision:

1."Although gap junctions are known to transmit calcium and second messengers, whether these signals causally instruct chemical synapse development-and how these transiently essential connections are themselves subsequently eliminated-remain entirely unknown." The cited citations have already implicated the hypothesis. I suggest to use "remain mechanistically unclear"

We thank the reviewer for this correction and have revised the text accordingly.

2. Figure 3D required a wild-type control for proper comparison.

We have added the wild-type control to Figure 3D.

3. The authors should also indicate N in every graph for the reader to easily access.

Sample sizes (N) have been added to the figure legend of every graph in the revised manuscript.

4. Page 9 "Remarkably, expression of UNC-76(S54E) in unc-76(e911) mutant worms partially rescued
This was indeed a typo—the correct strain should be unc-51(e1189). We have corrected this in the revised manuscript.

Reviewer #3:

Huang et al. demonstrate that fluorescence labeled innexin proteins form transient clusters on the PLM process during early development, which subsequently disappear as the animals mature. Disruption of these transient innexin clusters leads to a reduction in later-developing chemical synapses, accompanied by diminished high-frequency calcium oscillations. The authors further identify that UNC-51/ULK is required for this process. Previous studies have suggested that UNC-51 may regulate UNC-76/FEZ, which in turn links to UNC-69. Based on this, phenotypic analysis hinted that UNC-76 and UNC-69 may function in conjunction with UNC-51 to regulate the elimination of transient gap junctions.

Major Points:

1. Identification of transient vs. stable GJ partners: Neural circuit re-wiring during development is a well-established phenomenon in the C. elegans nervous system. The transiently formed innexin clusters described here likely represent neuronal connections with transient developmental partners. Moreover, gap junction (GJ) formation could be influenced by both sides of the partnering cells. Therefore, it is critical to determine whether the synaptic partners of PLM in early (L1) stages differ from those in later stages. If so, disruption of these transient innexin clusters may damage PLM-related connectivity indirectly-by affecting upstream developmental events such as cell migration or axon guidance-rather than directly impacting the formation of chemical synapses or calcium signals. To strengthen the significance of transient GJ elimination, the authors should identify the electrical synapse partners of PLM, distinguishing between stable connections (e.g., those near the cell body) and transient ones. Notably, GJ connections such as PLM-BDU have been previously characterized; similar experimental approaches could be employed to systematically identify those GJ partners of PLM.

We performed additional experiments to address the identity of transient versus stable gap junction partners in PLM neurons.

To test whether PLM's transient gap junctions require a partnering cell, we used a Cre-loxP-based cell-specific excision strategy to remove UNC-9 selectively from PLM neurons. Transient UNC-9 puncta persisted along the PLM process despite this excision, indicating that these puncta are supplied by a partnering cell rather than PLM alone, and confirming that they represent genuine intercellular gap junctions rather than intracellular aggregates. To identify the partner cell type, we examined co-localization of transient UNC-9 puncta with markers of different cell types. Transient puncta showed little co-localization with neuronal markers but partial co-localization with the hypodermal innexin INX-9, suggesting hypodermal cells as one class of transient partner; we were unable to functionally validate this (e.g., via calcium imaging in hypodermal cells), so we regard this evidence as suggestive rather than conclusive. Stable puncta persisting into L4 likely represent canonical neuron-neuron gap junctions, consistent with PLM's known interneuron partners (PVC,

LUA, PVR, BDU) from prior EM reconstruction; however, our imaging was centered on the PLM process near the cell body and did not extend to the region where these connections have been anatomically described, so we cannot confirm which specific partner corresponds to the stable puncta quantified here.

This distinction bears on two alternative explanations the reviewer raised for the pathway mutant phenotypes. First, if transient junctions are neuron-hypodermal rather than neuron-neuron, persistent junctions in pathway mutants cannot simply reflect a partner cell failing to migrate away, since the hypodermis is stationary. Second, regarding altered PLM axon guidance: *unc-51* mutants do show axon developmental abnormalities, so animals with overtly abnormal PLM axon morphology were excluded when scoring the excessive electrical synapse phenotype in this background. *unc-76* mutants, by contrast, showed no detectable PLM axon abnormality yet displayed the same excessive electrical synapse phenotype and failure of developmental elimination as *unc-51* mutants—arguing against altered axon guidance as the underlying cause. This concern could also extend to the partner neuron, since *unc-51* is broadly expressed and could in principle disrupt guidance in the partner cell rather than PLM itself; however, our finding that PLM-specific UNC-51 expression alone is sufficient to rescue the phenotype (Fig. 3B) demonstrates a cell-autonomous requirement within PLM, arguing against a defect originating from the partner neuron. Directly supporting the stationary-partner argument, persistent UNC-9 puncta in *unc-76* mutants continued to co-localize with hypodermal INX-9 at levels comparable to wild-type transient synapses (Fig. S3E), confirming that these puncta remain at their native hypodermal-apposed position rather than being redistributed to an ectopic site as a consequence of the mutant background.

2. Characterization of stabilized GJ in unc-51 and other mutants: The transient GJs appear abnormally stabilized in unc-51 mutants. Without identifying the specific PLM GJ partners in both wild-type and unc-51 mutant backgrounds, it remains unclear whether the abnormally stabilized GJ clusters in unc-51 mutants represent the same transient partnerships seen in wild-type animals or entirely different categories of junctions. This ambiguity undermines the core conclusion that UNC-51 is specifically involved in eliminating transient GJs. The same concern applies to analyses of unc-76, unc-69, and rab-10; the authors should identify the GJ partners of PLM under each genetic condition before drawing meaningful conclusions about their roles in GJ elimination.

To address this, we examined co-localization of persistent UNC-9 puncta with the hypodermal innexin INX-9 in *unc-76* mutants—the cleanest background for this analysis given its normal PLM axon morphology (Fig. S3E). Persistent puncta in *unc-76* mutants co-localized with hypodermal INX-9 at levels comparable to wild-type transient synapses, indicating that the stabilized junctions in this mutant represent the same hypodermal partnerships normally eliminated in wild-type, rather than a distinct category of junction. Moreover, developmental staging showed that *unc-76, unc-69,* and *rab-10* mutants each abolish the normal L1-to-L4 reduction in electrical synapse number (Fig. 3E,F; Fig. S6A,B), confirming that these mutants block the developmental elimination process itself rather than independently increasing gap junction number through an unrelated mechanism.

3. Clarification of UNC-51/UNC-76 genetic interaction: The proposed regulatory relationship between UNC-51 and UNC-76 is confusing and, in places, appears to conflict with the presented data. Although unc-76 mutants exhibit phenotypes similar to unc-51, the observation that UNC-76 expression is five-fold higher in unc-51 mutants than in wild-type directly contradicts the conclusion that UNC-51 acts upstream to positively regulate UNC-76 function in GJ elimination. Did the authors examine GJ phenotypes using a gain-of-function allele of unc-76? Alternatively-and perhaps more plausibly-UNC-76 and UNC-51 may act in parallel pathways or distinct biological processes to regulate GJ formation. This alternative interpretation should be considered and investigated.

Regarding the gain-of-function allele of *unc-76*: our phosphomimetic UNC-76(S54E) substitution effectively represents a gain-of-function approach, as it constitutively mimics the phosphorylated state of UNC-76. This approach was motivated by prior work in Drosophila demonstrating that UNC-51 directly phosphorylates UNC-76 at Ser143 to regulate motor-cargo assembly during axonal transport (Toda et al., 2008), and our identification of a conserved homologous phosphorylation site at Ser54 in the *C. elegans* UNC-76 long isoform. Critically, expression of UNC-76(S54E) in *unc-51* mutants partially rescued the excessive electrical synapse phenotype, whereas wild-type UNC-76 expression showed no rescue effect. This result demonstrates that phosphorylated UNC-76 is sufficient to partially bypass the requirement for UNC-51, providing direct evidence that UNC-76 functions downstream of UNC-51 in this process.

Regarding the apparent contradiction between UNC-51 acting upstream of UNC-76 and the five-fold upregulation of UNC-76 protein in *unc-51* mutants: we acknowledge that this observation requires explanation. We propose that elevated UNC-76 protein levels in *unc-51* mutants may reflect a compensatory response to the absence of functional phosphorylated UNC-76—when UNC-51-mediated phosphorylation is lost, the system may attempt to compensate by upregulating total UNC-76 protein abundance. However, this compensation appears ineffective: overexpression of wild-type UNC-76 alone does not rescue the *unc-51* phenotype, whereas phosphomimetic UNC-76(S54E) does. This indicates that it is specifically the phosphorylated form of UNC-76, rather than its total abundance, that is required for electrical synapse elimination. We acknowledge that the precise mechanism underlying UNC-76 upregulation in *unc-51* mutants remains to be determined.

Regarding the possibility that UNC-51 and UNC-76 act in parallel pathways: we cannot formally exclude this possibility. However, several lines of evidence support their functioning within the same genetic pathway. First, *unc-76* and *unc-51* single mutants display similar excessive electrical synapse phenotypes. Second, *unc-51;unc-76* double mutants do not show significantly enhanced defects compared to either single mutant alone, consistent with epistasis within a linear pathway. This non-additive relationship is not limited to the structural gap junction phenotype: wavelet analysis of calcium dynamics showed that *unc-51;unc-76* double mutants also exhibit elevated high-frequency energy proportion comparable to either single mutant alone, with no further enhancement (Fig. 3G,I), providing an independent, functional readout consistent with epistasis rather than parallel pathways. Third, as noted above, phosphomimetic UNC-76(S54E) partially rescues unc-51 mutants, placing UNC-76 functionally downstream of UNC-51. We note, however,

that the stronger phenotype of *unc-51* mutants relative to *unc-76* mutants and the incomplete rescue by UNC-76(S54E) together suggest that UNC-51 likely phosphorylates additional downstream targets beyond UNC-76 in this process, consistent with the known broad substrate repertoire of UNC-51/ULK1 across species (Toda et al., 2008; Russell et al., 2013; Papinski et al., 2014). We will discuss this possibility in the revised manuscript.

4. The phenotypic similarity alone is insufficient to support the proposed linear regulatory cascade from UNC-51 to UNC-76 to UNC-69 to RAB-10. The current data primarily rely on the so-called stabilized "transient gap junction" as a readout. While loss-of-function of the above genes all display similar phenotypes, this is not sufficient evidence to establish a sequential regulation cascade. More direct and insightful mechanistic links should be provided.

For UNC-51→UNC-76, we identified the specific phosphorylation site (Ser54) and showed that phosphomimetic UNC-76(S54E) rescues both the structural (excessive electrical synapse, Fig. 4G,H) and functional (elevated calcium energy proportion, Fig. 3I) phenotypes of *unc-51* mutants, while wild-type UNC-76 does not—a direct biochemical epistasis rather than mere phenotypic overlap. This is further supported by UNC-51/UNC-76 co-localization along the PLM axon (Fig. 4C, D) and by UNC-51 negatively regulating UNC-76 protein abundance (Fig. 4E,F). Neither *unc-51* nor *unc-76* loss-of-function altered the baseline ratio of retrograde to anterograde UNC-9 movements, whereas expression of phosphomimetic UNC-76(S54E) significantly increased this ratio in both wild-type and *unc-76* mutant backgrounds (Fig. 5C), directly demonstrating that the phosphorylated state of UNC-76—rather than loss of UNC-51 or UNC-76 function—drives the directional transport bias. For UNC-76→RAB-10→UNC-9, we directly visualized co-transport of UNC-76 with RAB-10-positive vesicles and of RAB-10-positive vesicles with UNC-9 at the single-vesicle level (Fig. 6C–G), together with non-additive genetic interaction between RAB-10(T23N) and *unc-51/unc-76* (Fig. 6A,B).

Minor Points:

1. Discrepancy in rescue experiment description: The description of the unc-76 rescue experiments is inconsistent and requires clarification. In the text, the authors state: "expression of UNC-76(S54E) in unc-76(e911) mutant worms partially rescued the excessive electrical synapse phenotypes, whereas wild-type UNC-76 expression showed no rescue effect (Fig. 4C,D)." However, Figures 4C and 4D indicate that unc-76 expression was actually performed in an unc-51 mutant background. This discrepancy must be resolved.

We thank the reviewer for catching this discrepancy. We apologize for the error. The rescue experiments shown in previous Figures 4C and 4D (now Figure 4G and 4H) were performed in the *unc-51(e1189)* mutant background, not *unc-76(e911)*. We have corrected the text accordingly.

2. Missing citations: The manuscript appears to omit several relevant citations, particularly previous findings on how gap junctions influence chemical synapse formation during early nervous system development, as well as established knowledge (and gaps in knowledge) regarding the GJ partners of

PLM in C. elegans. These references should be incorporated to properly contextualize the findings.

We have incorporated the relevant citations and included the corresponding background context accordingly.

September 9, 2026

RE: JCB Manuscript #202602115R

Lingfeng Meng
Zhejiang University-University of Edinburgh Institute

Dear Dr. Meng:

Thank you for submitting your revised manuscript entitled "Developmental Elimination of Electrical Synapses by UNC-51/UNC-76-Mediated Vesicular Transport". The reviewers all now support publication so we would be happy to publish your paper in JCB pending final revisions necessary to meet our formatting guidelines (see details below).

In your final revision, please be sure to address reviewer #1's final minor concerns.

To avoid unnecessary delays in the acceptance and publication of your paper, please read the following information carefully.

A. MANUSCRIPT ORGANIZATION AND FORMATTING:

Full guidelines are available on our Instructions for Authors page, http://jcb.rupress.org/submission-guidelines#revised.
Submission of a paper that does not conform to JCB guidelines will delay the acceptance of your manuscript.

1) Text limits: Character count for Articles is < 40,000, not including spaces. Count includes abstract, introduction, results, discussion, and acknowledgments. Count does not include title page, figure legends, materials and methods, references, tables, or supplemental legends.

2) Figures limits: Articles may have up to 10 main text figures.

3) Figure formatting: Scale bars must be present on all microscopy images, including inset magnifications. Molecular weight or nucleic acid size markers must be included on all gel electrophoresis. Aspect ratios of images may not be altered.

4) Statistical analysis: Error bars on graphic representations of numerical data must be clearly described in the figure legend. The number of independent data points (n) represented in a graph must be indicated in the legend. Statistical methods should be explained in full in the materials and methods. For figures presenting pooled data the statistical measure should be defined in the figure legends. Please also be sure to indicate the statistical tests used in each of your experiments (either in the figure legend itself or in a separate methods section) as well as the parameters of the test (for example, if you ran a t-test, please indicate if it was one- or two-sided, etc.). Also, if you used parametric tests, please indicate if the data distribution was tested for normality (and if so, how). If not, you must state something to the effect that "Data distribution was assumed to be normal but this was not formally tested."

5) Abstract and title: The abstract should be no longer than 160 words and should communicate the significance of the paper for a general audience. The title should be less than 100 characters including spaces. Make the title concise but accessible to a general readership.

6) Materials and methods: Should be comprehensive and not simply reference a previous publication for details on how an experiment was performed. Please provide full descriptions in the text for readers who may not have access to referenced manuscripts.

7) All antibodies, cell lines, animals, and tools used in the manuscript should be described in full, including accession numbers for materials available in a public repository such as the Resource Identification Portal. Please be sure to provide the sequences for all of your primers/oligos and RNAi constructs in the materials and methods. You must also indicate in the methods the source, species, and catalog numbers (where appropriate) for all of your antibodies. Please also indicate the acquisition and quantification methods for immunoblotting/western blots.

8) Microscope image acquisition: The following information must be provided about the acquisition and processing of images:
a. Make and model of microscope
b. Type, magnification, and numerical aperture of the objective lenses
c. Temperature
d. Imaging medium
e. Fluorochromes
f. Camera make and model
g. Acquisition software

h. Any software used for image processing subsequent to data acquisition. Please include details and types of operations involved (e.g., type of deconvolution, 3D reconstitutions, surface or volume rendering, gamma adjustments, etc.).

**9) References: There is no limit to the number of references cited in a manuscript. References should be cited parenthetically in the text by author and year of publication. Abbreviate the names of journals according to PubMed.

**10) Supplemental materials: There are strict limits on the allowable amount of supplemental data. Articles may have up to 5 supplemental figures. You currently have 6 supplemental figures, and we will be able to give you a bit more space in this case. Please also note that tables, like figures, should be provided as individual, editable files. A summary of all supplemental material should appear at the end of the Materials and methods section.

11) eTOC summary: A ~40-50-word summary that describes the context and significance of the findings for a general readership should be included on the title page. The statement should be written in the present tense and refer to the work in the third person.

12) Conflict of interest statement: JCB requires inclusion of a statement in the acknowledgements regarding competing financial interests. If no competing financial interests exist, please include the following statement: "The authors declare no competing financial interests." If competing interests are declared, please follow your statement of these competing interests with the following statement: "The authors declare no further competing financial interests."

**13) ORCID IDs: ORCID IDs are unique identifiers allowing researchers to create a record of their various scholarly contributions in a single place. Please note that ORCID IDs are now *required* for all authors. At resubmission of your final files, please be sure to provide your ORCID ID and those of all co-authors.

14) A separate author contribution section following the Acknowledgments. All authors should be mentioned and designated by their full names. We encourage use of the CRediT nomenclature.

Please note that JCB now requires authors to submit Source Data used to generate figures containing gels and Western blots with all revised manuscripts. This Source Data consists of fully uncropped and unprocessed images for each gel/blot displayed in the main and supplemental figures. For assays performed using capillary electrophoresis and/or immunoassay-based detection, authors should instead provide the electropherogram graph(s) for each experiment, plotting fluorescence/chemiluminescence intensity vs. molecular weight/size. Please be sure to provide one Source Data file for each figure gels, blots, and/or capillary electrophoresis assays along with your revised manuscript files. File names for Source Data figures should be alphanumeric without any spaces or special characters (i.e., SourceDataF#, where F# refers to the associated main figure number or SourceDataFS# for those associated with Supplementary figures). For traditional gels and blots, the lanes of the gels/blots should be labeled as they are in the associated figure, the place where cropping was applied should be marked (with a box), and molecular weight/size standards should be labeled wherever possible. For capillary electrophoresis assays, each trace in the graph should be color-coded and labeled to indicate which protein, gene, or sample is being measured (please try to avoid red/green combinations to accommodate our color-blind readers).

Source Data files will be made available to reviewers during evaluation of revised manuscripts and, if your paper is eventually published in JCB, the files will be directly linked to specific figures in the published article.

Source Data Figures should be provided as individual PDF files (one file per figure). Authors should endeavor to retain a minimum resolution of 300 dpi or pixels per inch. Please review our instructions for export from Photoshop, Illustrator, and PowerPoint here: https://rupress.org/jcb/pages/submission-guidelines#revised

Journal of Cell Biology now requires a data availability statement for all research article submissions. These statements will be published in the article directly above the Acknowledgments. The statement should address all data underlying the research presented in the manuscript. Please visit the JCB instructions for authors for guidelines and examples of statements at (https://rupress.org/jcb/pages/editorial-policies#data-availability-statement).

B. FINAL FILES:

Please upload the following materials to our online submission system. These items are required prior to acceptance. If you have any questions, contact JCB's Managing Editor, Lindsey Hollander (lhollander@rockefeller.edu).

-- An editable version of the final text (.DOC or .DOCX) is needed for copyediting (no PDFs).

-- High-resolution figure and MP4 video files: See our detailed guidelines for preparing your production-ready images, https://jcb.rupress.org/fig-vid-guidelines.

-- Cover images: If you have any striking images related to this story, we would be happy to consider them for inclusion on the

journal cover. Submitted images may also be chosen for highlighting on the journal table of contents or JCB homepage carousel. Images should be uploaded as TIFF or EPS files and must be at least 300 dpi resolution.

It is JCB policy that if requested, original data images must be made available to the editors. Failure to provide original images upon request will result in unavoidable delays in publication. Please ensure that you have access to all original data images prior to final submission.

The license to publish form must be signed before your manuscript can be sent to production. A link to the license to publish form will be sent to the corresponding author only. Please take a moment to check your funder requirements before choosing the appropriate license.

Additionally, JCB encourages authors to submit a short video summary of their work. These videos are intended to convey the main messages of the study to a non-specialist, scientific audience. Think of them as an extended version of your abstract, or a short poster presentation. We encourage first authors to present the results to increase their visibility. The videos will be shared on social media to promote your work. For more detailed guidelines and tips on preparing your video, please visit https://rupress.org/jcb/pages/submission-guidelines#videoSummaries.

Thank you for your attention to these final processing requirements. Please revise and format the manuscript and upload materials within 14 days. If you need an extension for whatever reason, please let us know and we can work with you to determine a suitable revision period.

Please contact the journal office with any questions at cellbio@rockefeller.edu.

Thank you for this interesting contribution, we look forward to publishing your paper in Journal of Cell Biology.

Sincerely,

Xiang Yu
Monitoring Editor
Journal of Cell Biology

Gabriele Stephan
Scientific Editor
Journal of Cell Biology

Reviewer #1:

The authors have addressed most of my concerns. However, there are still a few points that need to be clarified or discussed.
1. Fig. S1E. The experimental logic is not easy to follow. This experiment appears to show that UNC-9 at these puncta is contributed by PLM, but does not necessarily establish that the partnering cell is non-neuronal, as a neuronal partner could use a different innexin to form a heterotypic gap junction. Please clarify the logic and revise the conclusion accordingly.

2. "However, wavelet transform analysis of calcium dynamics revealed that L1 neurons exhibited significantly higher energy proportion (48.8%) compared to L4 (31.0%) (Fig. 2B,E). ..., indicating that electrical synapses enhance highfrequency calcium oscillations during early development.."
The conclusion that "electrical synapses enhance high-frequency calcium oscillations" is stronger than supported by the data. The L1-L4 difference demonstrates a developmental change in high-frequency activity, but does not establish its dependence on electrical synapses. Please revise the conclusion unless direct evidence supports this causal relationship.

3. The statement that premature elimination of transient electrical synapses disrupts chemical synapse formation is not fully supported, as the miniSOG manipulation appears to eliminate UNC-9 broadly, including stable junctions. Please revise the conclusion.

4. Fig. 4I. The unc-51; unc-76 double mutant appears to show a stronger phenotype than either single mutant. Please directly compare the double and single mutants statistically and reconsider the genetic interpretation if an additive effect is present.

5. The unchanged retrograde/anterograde ratio in unc-76 mutants does not establish that unphosphorylated UNC-76 is "direction-neutral," since the mutant removes both phosphorylated and unphosphorylated UNC-76. Likewise, UNC-76(S54E) supports sufficiency of the phosphomimetic form, but not the requirement for endogenous UNC-51-dependent phosphorylation. Please clarify and temper this interpretation.

6. The developmental change in UNC-51 localization is informative, but does not fully explain why electrical synapse elimination occurs specifically at L1. In particular, what regulates UNC-51 localization/activity at transient junctions remains unclear. This limitation should at least be acknowledged and discussed.

Reviewer #2:

The authors have done a good job in addressing not only our comments, but also comments by other reviewers and I believe that the manuscript requires no further revision before publication.

Reviewer #3:

The authors addressed questions and concerns raised by this reviewer satisfactorily.

Reviewer #1:

The authors have addressed most of my concerns. However, there are still a few points that need to be clarified or discussed.

1. Fig. S1E. The experimental logic is not easy to follow. This experiment appears to show that UNC-9 at these puncta is contributed by PLM, but does not necessarily establish that the partnering cell is non-neuronal, as a neuronal partner could use a different innexin to form a heterotypic gap junction. Please clarify the logic and revise the conclusion accordingly.

We agree with the reviewer that we cannot formally exclude a neuronal partner coupling to PLM UNC-9 through a heterotypic channel with a different innexin. However, our experience with these reporters is that an exogenously expressed innexin traffics to and accumulates at sites where a partner cell has the structural capacity to form a gap junction, even if the corresponding endogenous innexin is not itself enriched there. For example (data not shown), exogenously expressed INX-3 in PLM localizes to puncta at the tail region coincident with the PVC/LUA/PVR synaptic sites in adults, even though C-terminally tagged endogenous INX-3 shows no detectable expression in PLM in adults. We therefore believe this assay still offers a useful, if not definitive, clue to partner identity.

We have revised the text to state only that we used the innexin most highly expressed in each candidate tissue, softened the conclusion to a tentative possibility rather than an exclusion, and restructured the sentence for clarity.

2. "However, wavelet transform analysis of calcium dynamics revealed that L1 neurons exhibited significantly higher energy proportion (48.8%) compared to L4 (31.0%) (Fig. 2B,E). ..., indicating that electrical synapses enhance highfrequency calcium oscillations during early development.."

The conclusion that "electrical synapses enhance high-frequency calcium oscillations" is stronger than supported by the data. The L1-L4 difference demonstrates a developmental change in high-frequency activity, but does not establish its dependence on electrical synapses. Please revise the conclusion unless direct evidence supports this causal relationship.

We agree that the L1-versus-L4 comparison alone is correlative. We have softened this sentence to raise it only as a possibility.

3. The statement that premature elimination of transient electrical synapses disrupts chemical synapse formation is not fully supported, as the miniSOG manipulation appears to eliminate UNC-9 broadly, including stable junctions. Please revise the conclusion.

We apologize for the lack of clarity. Our spinning disk confocal system is equipped with a Live-SR module incorporating a steerable prism that restricts the excitation beam to a user-defined region of interest, allowing us to confine blue-light illumination to the distal, transient-synapse-containing region of the PLM neurite while sparing the stable junctions near the cell body. We will

describe this in greater detail in the Methods section. Consistent with this, in a parallel experiment using comparable targeted illumination (Dendra2::UNC-9 photoactivation), the stable synapses near the cell body and the soma itself were completely unaffected (Fig. 1J,K and S1K,L) — this is direct internal evidence addressing exactly the concern raised here, not new data but reuse of a control we had already shown. Sorry for the confusion.

4. Fig. 4I. The unc-51; unc-76 double mutant appears to show a stronger phenotype than either single mutant. Please directly compare the double and single mutants statistically and reconsider the genetic interpretation if an additive effect is present.
We believe the reviewer is referring to Fig. 3I, as Fig. 4I addresses an unrelated experiment. We compared the double mutant directly to each single mutant using Welch's ANOVA with Dunnett's T3 multiple comparisons test. No significant difference was found between the *unc-51;unc-76* double mutant and either the *unc-51* ($p = 0.4168$) or *unc-76* ($p = 0.9432$) single mutant, indicating no additive effect and supporting our conclusion that UNC-51 and UNC-76 act within the same genetic pathway. We have added these comparisons to Fig. 3I and updated the Methods accordingly.

5. The unchanged retrograde/anterograde ratio in unc-76 mutants does not establish that unphosphorylated UNC-76 is "direction-neutral," since the mutant removes both phosphorylated and unphosphorylated UNC-76. Likewise, UNC-76(S54E) supports sufficiency of the phosphomimetic form, but not the requirement for endogenous UNC-51-dependent phosphorylation. Please clarify and temper this interpretation.
We agree and have tempered the interpretation: we no longer describe unphosphorylated UNC-76 as "direction-neutral" (the null mutant cannot isolate that specific contribution), and we now present the phosphorylation-triggered transition as a proposed model rather than an established event.

6. The developmental change in UNC-51 localization is informative, but does not fully explain why electrical synapse elimination occurs specifically at L1. In particular, what regulates UNC-51 localization/activity at transient junctions remains unclear. This limitation should at least be acknowledged and discussed.
We agree this is an important point. We believe UNC-51 acts downstream of an upstream developmental cue that restricts its localization/activity to transient junctions at L1, though the specific identity of this cue remains unclear. We have added this discussion to the manuscript.

Reviewer #2:

The authors have done a good job in addressing not only our comments, but

also comments by other reviewers and I believe that the manuscript requires no further revision before publication.

Reviewer #3:

The authors addressed questions and concerns raised by this reviewer satisfactorily.
